# MARK4 controls ischaemic heart failure through microtubule detyrosination

**DOI:** 10.1038/s41586-021-03573-5

**Published:** 2021-05-26

**Authors:** Xian Yu, Xiao Chen, Mamta Amrute-Nayak, Edward Allgeyer, Aite Zhao, Hannah Chenoweth, Marc Clement, James Harrison, Christian Doreth, George Sirinakis, Thomas Krieg, Huiyu Zhou, Hongda Huang, Kiyotaka Tokuraku, Daniel St Johnston, Ziad Mallat, Xuan Li

**Affiliations:** 1 Department of Medicine, Cardiovascular Division, University of Cambridge, Level 5, Box 157, Addenbrookes Hospital, Cambridge, UK, CB2 0QQ; 2 Department of Cardiology, Union Hospital, Tongji Medical College, Huazhong University of Science and Technology, Wuhan, China, 430030; 3 Department of Molecular and Cell physiology, Hannover Medical School, OE4210, Carl-Neuberg-Str.1, 30625 Hannover, Germany; 4 The Gurdon Institute and Department of Genetics, University of Cambridge, Tennis Court Road, Cambridge, UK, CB2 1QN; 5 College of Computer Science and Technology, QingDao University, 308 Ningxia Road, Shinan District, Shandong, China, 266071; 6 Department of Medicine, Experimental Medicine and Immunotherapeutics Division, University of Cambridge, Addenbrookes Hospital, Cambridge, UK, CB2 0QQ; 7 School of Informatics, University of Leicester, University Road, Leicester, UK, LE1 7RH; 8 Department of Biology, Southern University of Science and Technology, Shenzhen, China, 518055; 9 Muroran Institute of Technology, 27-1 Mizumoto-cho, Muroran 050-8585, Japan; 10 Université de Paris, Institut National de la Santé et de la Recherche Médicale, U970, PARCC, Paris, France

## Abstract

Myocardial infarction (MI) is a major cause of premature adult death. Compromised cardiac function after MI leads to chronic heart failure with systemic health complications and high mortality rate^1^. Effective therapeutic strategies are highly needed to improve the recovery of cardiac function after MI. More specifically, there is a major unmet need for a new class of drugs that improve cardiomyocyte contractility, because currently available inotropic therapies have been associated with high morbidity and mortality in patients with systolic heart failure^2,3^, or have shown a very modest risk reduction^4^. Microtubule detyrosination is emerging as an important mechanism of regulation of cardiomyocyte contractility^5^. Here, we show that deficiency of Microtubule-Affinity Regulating Kinase 4 (MARK4) substantially limits the reduction of left ventricular ejection fraction (LVEF) after acute MI in mice, without affecting infarct size or cardiac remodeling. Mechanistically, we provide evidence that MARK4 regulates cardiomyocyte contractility through promoting microtubule-associated protein 4 (MAP4) phosphorylation, thereby facilitating the access of Vasohibin 2 (VASH2), a tubulin carboxypeptidase (TCP), to microtubules for α-tubulin detyrosination. Our results show how cardiomyocyte microtubule detyrosination is finely tuned by MARK4 to regulate cardiac inotropy, and identify MARK4 as a promising druggable therapeutic target for improving cardiac function after MI.

Myocardial infarction, the main cause of ischaemic heart disease (IHD) and chronic heart failure, is a serious ischaemic syndrome in which the blood supply to the heart is blocked, thus causing substantial myocardial cell death and loss of function in the remaining viable cells^6^. Microtubule (MT) detyrosination, which is associated with DESMIN at force-generating sarcomeres^5^, is upregulated in the failing hearts of patients with ischaemic cardiomyopathy^5,7^ and hypertrophic cardiomyopathies^5,7,8^, and suppression of microtubule detyrosination improves contractility in failing cardiomyocytes^7^. VASH1 or VASH2, coupled with a small vasohibin-binding protein (SVBP), forms TCP that are capable of tubulin detyrosination^9,10^. Depletion of VASH1 speeds contraction and relaxation in failing human cardiomyocytes^11^. Structural and biophysical studies have suggested that VASH interacts with the C-terminal tail of α-tubulin^12-14^. However, the regulatory mechanisms of this system are still poorly understood.

Microtubule stability is regulated by microtubule-associated proteins (MAPs), including classical MAPs such as MAP2, MAP4, and Tau^15^. MAP4 is expressed in the cardiomyocytes and MAP4 level significantly increases in human hearts with cardiomyopathy^7^. MAP4 dephosphorylation on microtubule network has been described in a feline model of pressure overload cardiac hypertrophy^16^, but the relationship of MAP4 phosphorylation with microtubule detyrosination has not been examined. MARK4 is an evolutionarily conserved serine-threonine kinase^17,18^ known to phosphorylate MAPs including Tau, MAP2 and MAP4, on KXGS motif within their microtubule-binding motif^19-21^. The phosphorylation of MAPs triggered by MARK induces conformational changes that alter MAPs association with microtubules, and thereby regulates microtubule dynamics^19-21^. MARK4 is expressed in the hearts^20^, however the role of MARK4 in the cardiomyocyte has not been studied. Here, we examined whether MARK4 regulates the function of the failing cardiomyocyte through modulation of microtubule detyrosination.

## Heart function of *Mark4* ^-/-^ mice post-MI

**Extended Data Fig. 1:**
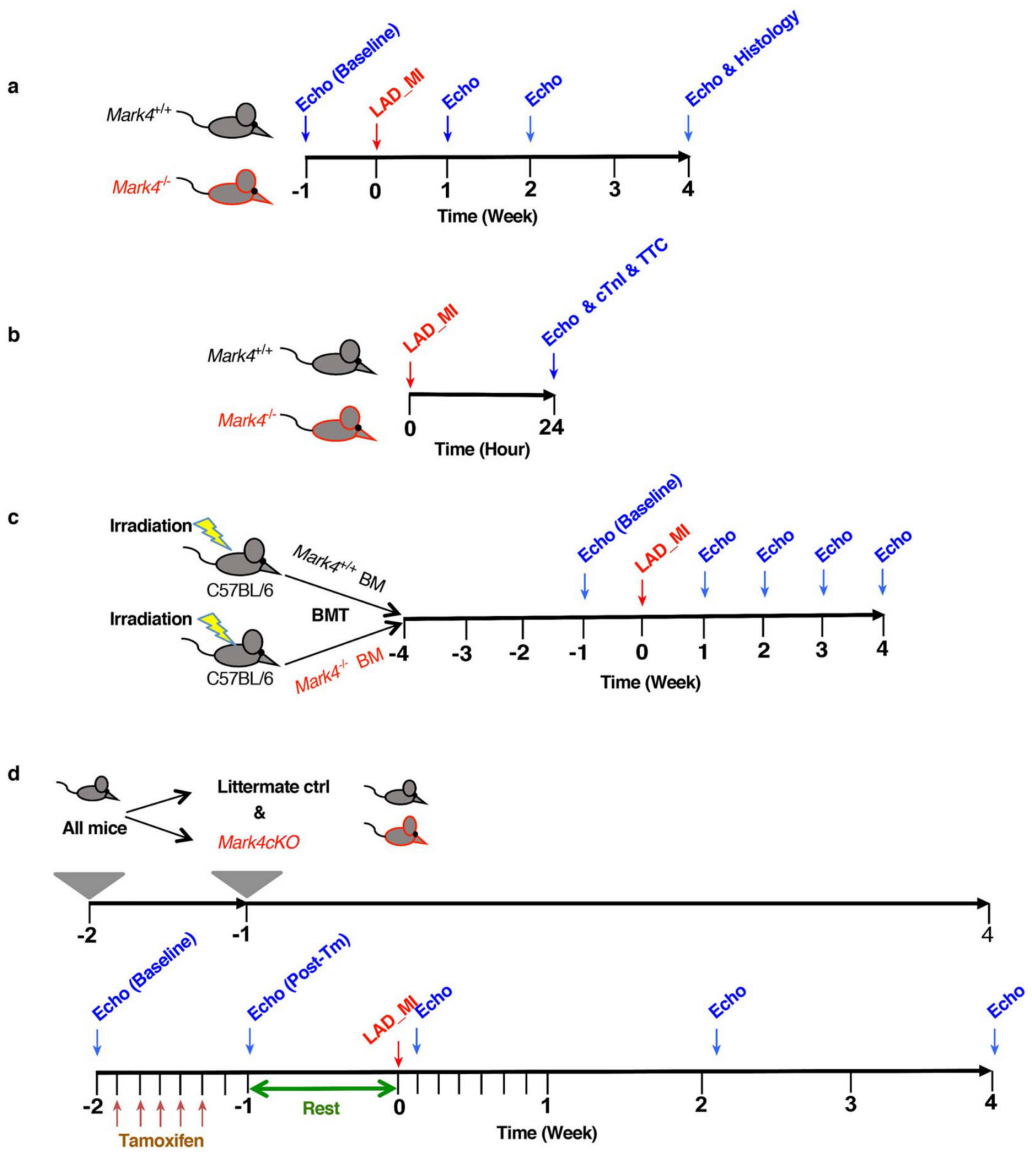
Timeline of experimental design. **a,** Timeline of experimental design for Fig. 1d and 1e. Investigation of the effect of total MARK4 deficiency on cardiac function using the model of left anterior descending (LAD) coronary artery ligation model to induce myocardial infarction (MI). Echocardiography (Echo) and histological analysis at the indicated time points. **b**, Timeline of experimental design for Fig. 2a, 2b, and 2c. Investigation of the effect of total MARK4 deficiency on cardiac function at 24 hours post-MI. Echocardiography, circulating cardiac troponin (cTnI), and histological analyses were performed at the indicated time point. **c**, Timeline of experimental design for Fig. 2d. Investigation of the effect of MARK4 expression in haematopoietic cells on cardiac function using the LAD ligation model. BM: bone marrow. BMT: bone marrow transplantation. Echocardiography analysis was performed at the indicated time points. **d**, Timeline of experimental design for Fig. 2e. Investigation of the effect of MARK4 expression in cardiomyocytes on cardiac function using the LAD ligation model. Tm: tamoxifen. *Mark4cKO*: conditional *Mark4* knock-out mice. Echocardiography analysis was performed at the indicated time points.

**Extended Data Fig. 2:**
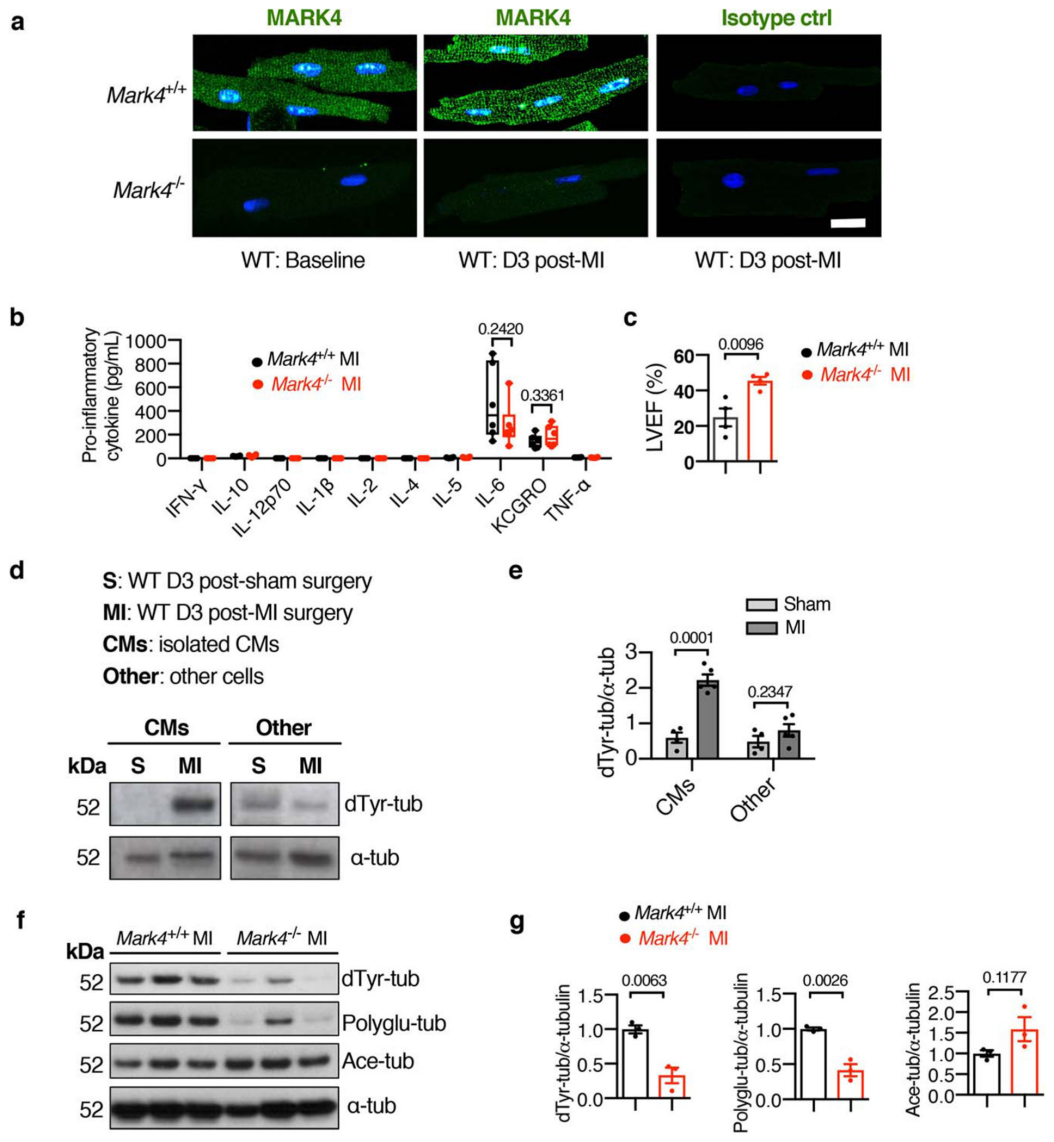
MARK4 expression, α-tubulin post-translational modifications, and changes in the inflammatory response post-myocardial infarction. **a**, Representative confocal images of primary cardiomyocytes (CMs) isolated from *Mark4* ^-/-^ or control mice at baseline (BL) or at day 3 post-MI (MI), scale bar= 20 μm. **b-c**, Levels of pro-inflammatory cytokines at day 3 post-MI (n=6 per group) (**b**). Left ventricular ejection fraction (LVEF) at day 3 post-MI (n=4 per group) (**c**). **d-e**, Western blots (WBs) of detyrosinated α-tubulin (dTyr-tub) in cell lysates of CMs isolated from wild-type mice at day 3 post-MI or post-sham surgery (S), with the lysates of the remaining cells from the same hearts used as control. Representative WBs (**d**). Ratio of dTyr-tubulin over total α-tubulin quantified using western blot data from biologically independent samples (S group: n=4 mice; MI group: n=5 mice) (**e**). **f-g**, Western blots of cell lysates from the isolated cardiomyocytes of *Mark4* ^-/-^ or control mice at day 3 post-MI, to detect detyrosinated α-tubulin (dTyr-tub), polyglutamylated α-tubulin (Polyglu-tub), acetylated α-tubulin (Ace-tub), and a–tubulin (α-tub). Representative images (**f**). Ratio of dTyr-tub, or polyglu-tub, or ace-tub over total α-tubulin quantified using western blot data from biologically independent samples (n=3 mice per group) (**g**). The box bounds represent the 25^th^ and 75^th^ percentiles, the middle line shows the median, and the whiskers show the minimum and maximum (**b**). Mean±s.e.m.; two-tailed unpaired *t*-test (**c, e, g**). *P* values are indicated on the graphs.

**Fig. 1:**
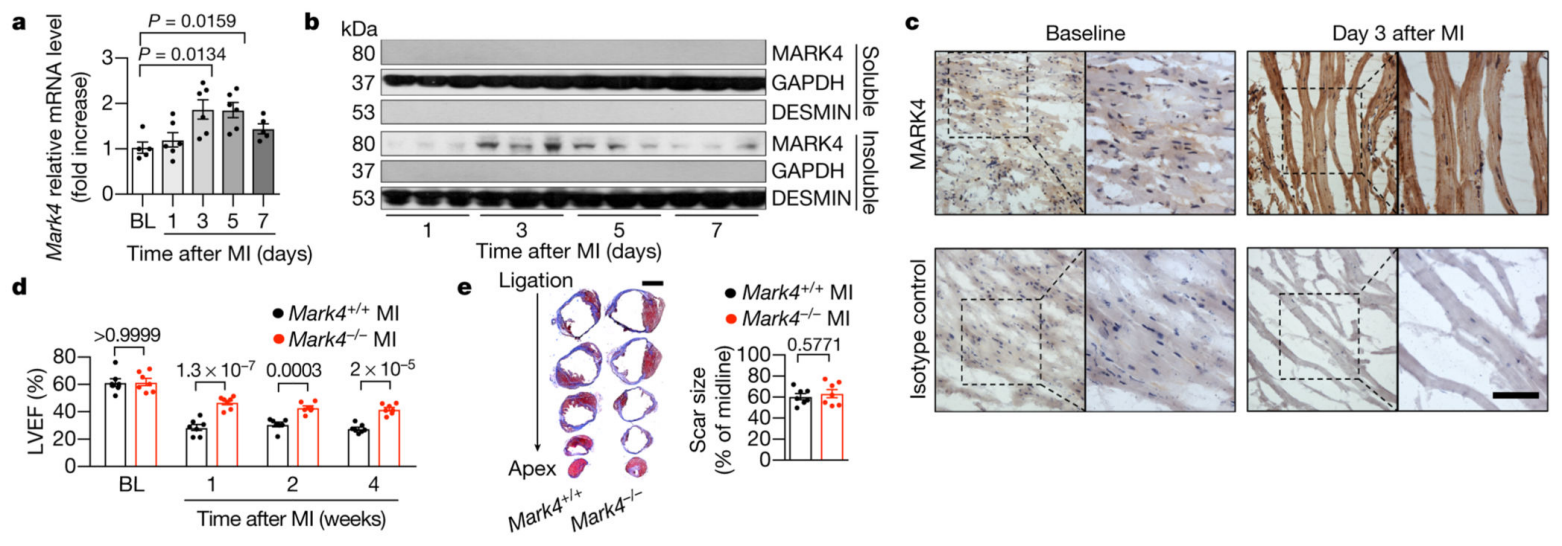
MARK4 deficiency preserves cardiac function after myocardial infarction without altering scar size. **a,** Real-time PCR of wild-type (WT) heart samples. Baseline (BL): heart without myocardial infarction (MI). D1, D3, D5, D7: hearts at the relevant days post-MI. n=5 at baseline, n=6 mice per time point at D1, D3 and D5 post-MI, and n=5 mice at D7 post-MI. **b,** Western blots of wild-type hearts post-MI. MARK4 in the insoluble (Ins.) cytoskeletal fractions (with DESMIN as marker), and GAPDH in corresponding soluble (S.) cytosolic fractions are shown. n=3 mice at each time point. **c,** Representative immunohistochemical staining of MARK4 in wild-type mice at baseline or post-MI. Isotype IgG was used as control. Scale bar=50μm. **d-e**, Assessment of left ventricular ejection fraction (LVEF) in *Mark4* ^-/-^ mice (n=7) and their littermate controls (*Mark4* ^+/+^) (n=7) at baseline, and at week 1 (W1), week 2 (W2), and week 4 (W4) post-MI (**d**). Scar size at week 4 post-MI (scale bar=2mm) (**e**). Mean ± s.e.m.; one-way ANOVA test with Bonferroni post-hoc correction (**a**); two-way ANOVA with Bonferroni post-hoc correction for multiple comparisons (**d**); two-tailed unpaired *t*-test (**e**). *P* values are indicated on the graphs.

To evaluate the effect of MARK4 in the setting of IHD, we used a murine model of permanent left anterior descending (LAD) coronary artery ligation to induce a large MI^22,23^(Extended Data Fig.1a). We detected *Mark4* mRNA (Fig.1a) and MARK4 protein (Fig.1b) expression in the heart tissues, peaking between day 3 and day 5 post-MI (Fig.1a-1c). MARK4 was almost exclusively detected in the cytoskeleton-enriched insoluble fraction of the whole heart extracts (Fig.1b), and was localized in the cardiomyocytes (Fig.1c; Extended Data Fig.2a). MARK4 deficient mice (*Mark4* ^-/-^) displayed a remarkable preservation of LVEF, which was 63.6% (± 5.8 %) higher compared with their wild-type littermate controls on the first week post LAD surgery (Fig.1d), without any alteration of cardiac remodeling (Supplementary Table1). Interestingly, infarct scar size was similar between the two groups of mice (Fig.1e), indicating that the substantial difference in cardiac function between wild-type and *Mark4* ^-/-^ mice was not attributable to differences of size in viable cardiac tissues.

## MARK4 regulates cardiac contractility

**Extended Data Fig. 3:**
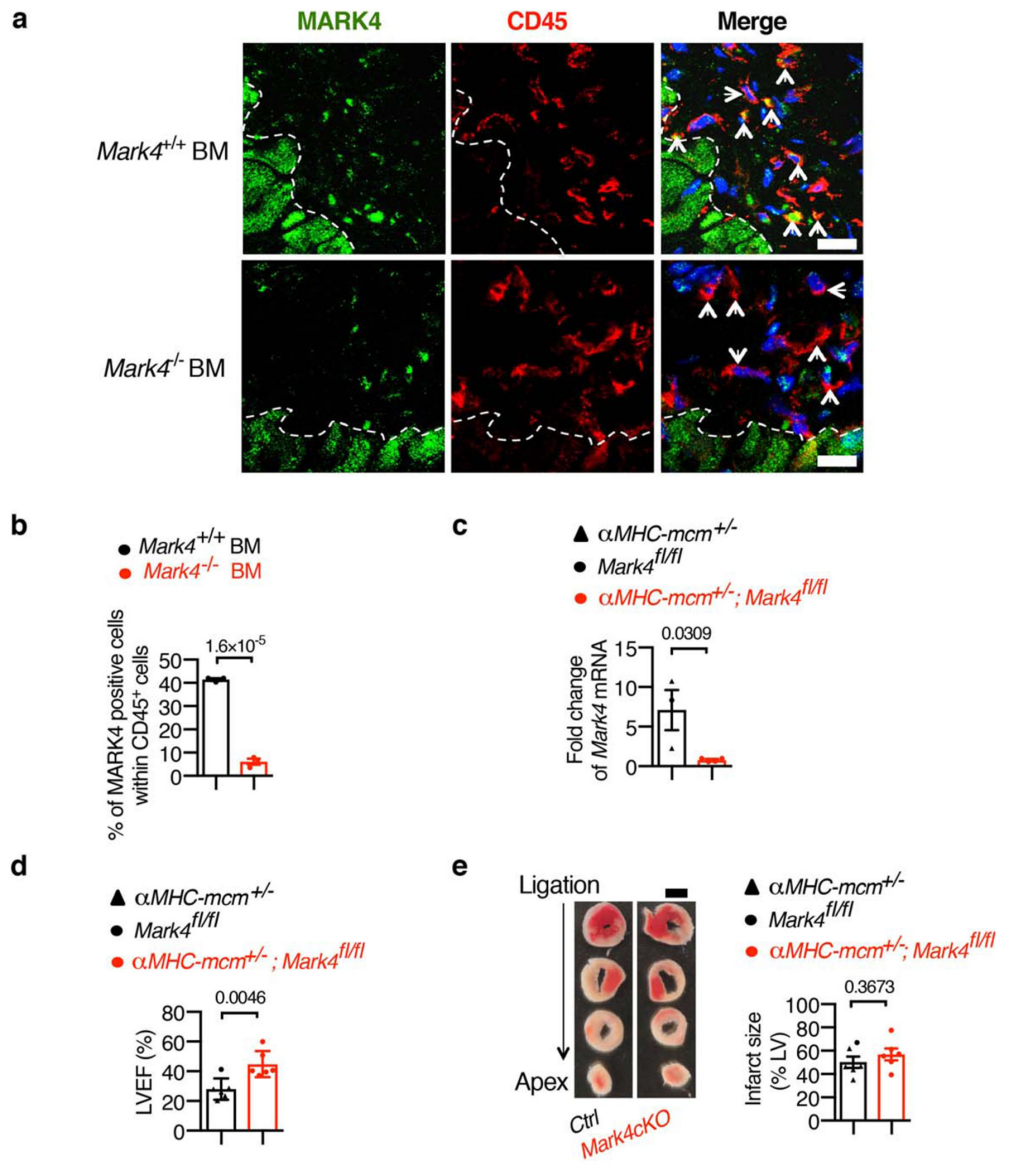
Validation of the murine models for MARK4 selective expression in either haematopoietic cells or cardiomyocytes. **a-b**, Confirmation of MARK4 deficiency in CD45^+^ cells of chimeric wild-type mice reconstituted with bone marrow (BM) cells from *Mark4* ^-/-^ mice(strategy in Extended Data Fig.1c). Representative image with arrows pointing to CD45^+^ cells in the infarct area, scale bar= 20 μm (**a**). Quantification of percentage of MARK4 positive cells (green) within CD45^+^ cells (red) (n=3 mice per group) (**b**). **c**, Confirmation of *Mark4* deletion in cardiomyocytes (strategy in Extended Data Fig.1d). Real-time PCR of *Mark4* level from primary cardiomyocytes isolated from *aMHC-mcm^+/-^;Mark4^fl/fl^* (n=4) and control mice (n=3) at day 7 post the last tamoxifen injection. **d-e**, Assessment of left ventricular ejection fraction (LVEF) of a different batch (from Fig. 2e) of conditional *Mark4* deficiency in cardiomyocytes (*Mark4*cKO) and control mice (n=6 per group) at day 1 post-myocardial infarction (MI) (**d**). Infarct size at 24 hours post-myocardial infarction (scale bar=2mm) (**e**). Mean±s.e.m.; two-tailed unpaired *t*-test (**b, c, d, e**). *P* values are indicated on the graphs.

**Fig. 2:**
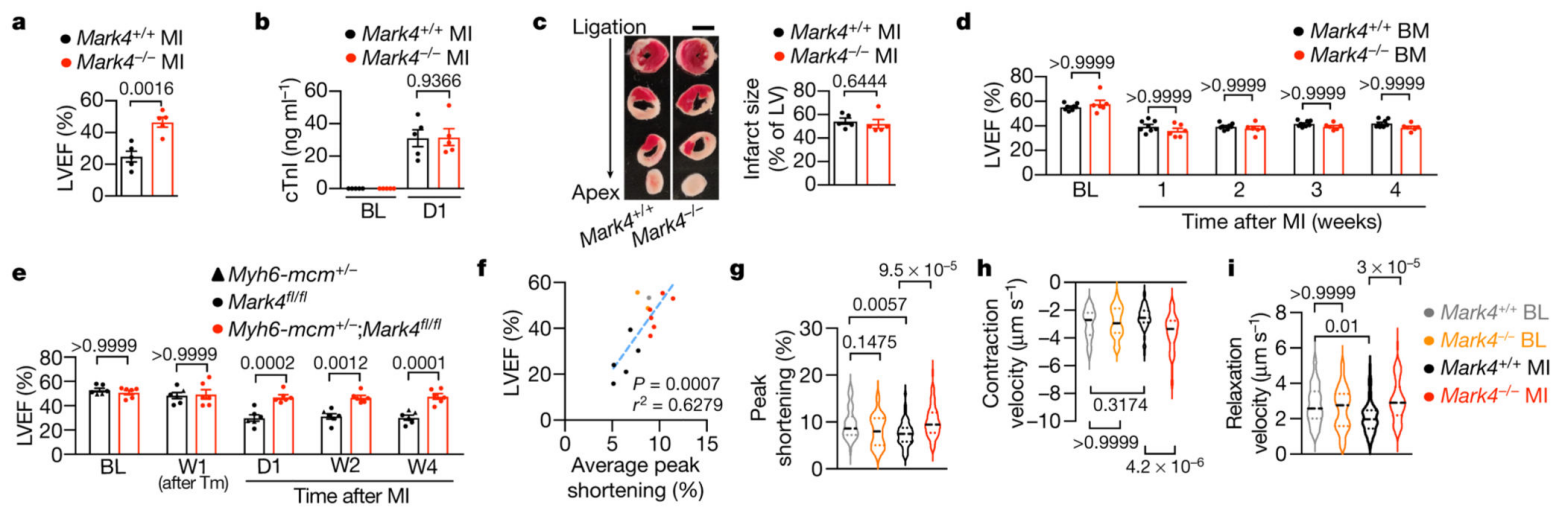
MARK4 expression in cardiomyocytes regulates cardiac contractile function after myocardial infarction. **a-c**, *Mark4* ^-/-^ mice (n=5) and their littermate controls (*Mark4* ^+/+^, n=5) at day 1 post-myocardial infarction (MI). Left ventricular ejection fraction (LVEF) (**a**). Circulating cardiac troponin I (cTnI) levels (**b**) and infarct size (scale bar=2mm) (**c**) at 24 hours (D1) post-MI are shown. cTnI measurements at Baseline (BL) were used as controls. **d**, Assessment of LVEF in chimeric mice (n=8 wild-type recipients of *Mark4* ^+/+^ bone marrow (BM) donors; n=6 wild-type recipients of *Mark4* ^-/-^ BM donors) at the indicated time points. **e**, Assessment of LVEF in conditional *Mark4* deficiency in *aMHC-mcm^+/-^;Mark4^fl/fl^* mice (n=6), with conditional *Mark4* deficiency induced by tamoxifen (Tm), at the indicated time points. Tamoxifen-injected littermate mice, *aMHC-mcm* ^+/-^ and *Mark4^fl/fl^*, were used as controls (n=6). **f-n**, Contractility assay of single primary cardiomyocytes (CMs) isolated at baseline (BL) or at day 3 post-MI in the following groups: *Mark4* ^+/+^ BL (n=4 mice / n=45 CMs examined over 4 independent experiments), *Mark4* ^-/-^ BL (n=3 mice / n=45 CMs examined over 3 independent experiments), *Mark4* ^+/+^ MI (n=5 mice / n=54 CMs examined over 5 independent experiments), and *Mark4* ^-/-^ MI (n=6 mice / n=57 CMs examined over 6 independent experiments). Colour denotation of samples (**f**). Correlation between LVEF (measured at day 1 post-MI) and sarcomere peak shortening (**g**). Sarcomere peak shortening (**h**). Pooled data of contraction velocity (**i**) and relaxation (**j**) velocity. Violin plots lines at the median and quartiles (**h-j**). Mean ± s.e.m.; two-tailed unpaired *t*-test (**a, b, c**); two-way ANOVA with Bonferroni post-hoc correction for multiple comparisons (**d, e, h, i, j**). *P* values are indicated on the graphs.

We found that the protective effect of MARK4 deficiency on the preservation of cardiac function was already apparent at 24 hours post-MI (Extended Data Fig.1b; Fig.2a), despite similar extent of myocardial injury, shown by comparable serum cardiac troponin I (cTnI) level (Fig.2b), and comparable infarct size analyzed by triphenyltetrazolium chloride (TTC) staining (Fig.2c), in *Mark4* ^-/-^ and wild-type mice. MARK4 has previously been shown to regulate NLRP3 activation in macrophages^24,25^, which could affect the outcome of post-ischaemic injury given the role of NLRP3 inflammasome in this setting^26,27^. However, MARK4 deficiency did not significantly alter local and systemic inflammatory responses to myocardial injury at day 3 post-MI (Supplementary Table 2; Extended Data Fig.2b) when the preservation of LVEF was already evident in *Mark4* ^-/-^ mice (Extended Data Fig.2c). Moreover, bone marrow transfer of *Mark4* ^-/-^ haematopoietic cells into wild-type mice (Extended Data Fig.1c; validation in Extended Data Fig.3a-3b) did not improve cardiac function after MI in comparison with the transfer of wild-type bone marrow cells (Fig.2d), indicating that the protective effect of MARK4 deficiency post-MI cannot be explained by the role of MARK4 in haematopoietic cells. In contrast, using an inducible conditional deletion of *Mark4* in cardiomyocytes (*Mark4cKO*) (Extended Data Fig.1d; validation in Extended Data Fig.3c), we found a substantial preservation of LVEF in *Mark4cKO* mice post-MI, which was 56.8% (± 6.2%) higher when compared with their littermate control mice at day one post-MI (Fig.2e). The protective effect seen in *Mark4cKO* started as early as the first day after MI and lasted until the end of the observation at four weeks post-MI (Fig.2e). Very impressively, *Mark4cKO* mice had only 4.3% (±3.8%) LVEF reduction at day one post-MI, as compared with 37.9% (±5.5 %) LVEF reduction in the control mice (Fig.2e), without any difference in infarct size (Extended Data Fig.3e). The data further show an impact of the remaining/viable MARK4-deficient cardiomyocytes on the contractile function. Collectively, our data demonstrate an intrinsic role of cardiomyocyte-expressed MARK4 in controlling cardiac function post-MI.

**Extended Data Fig. 4:**
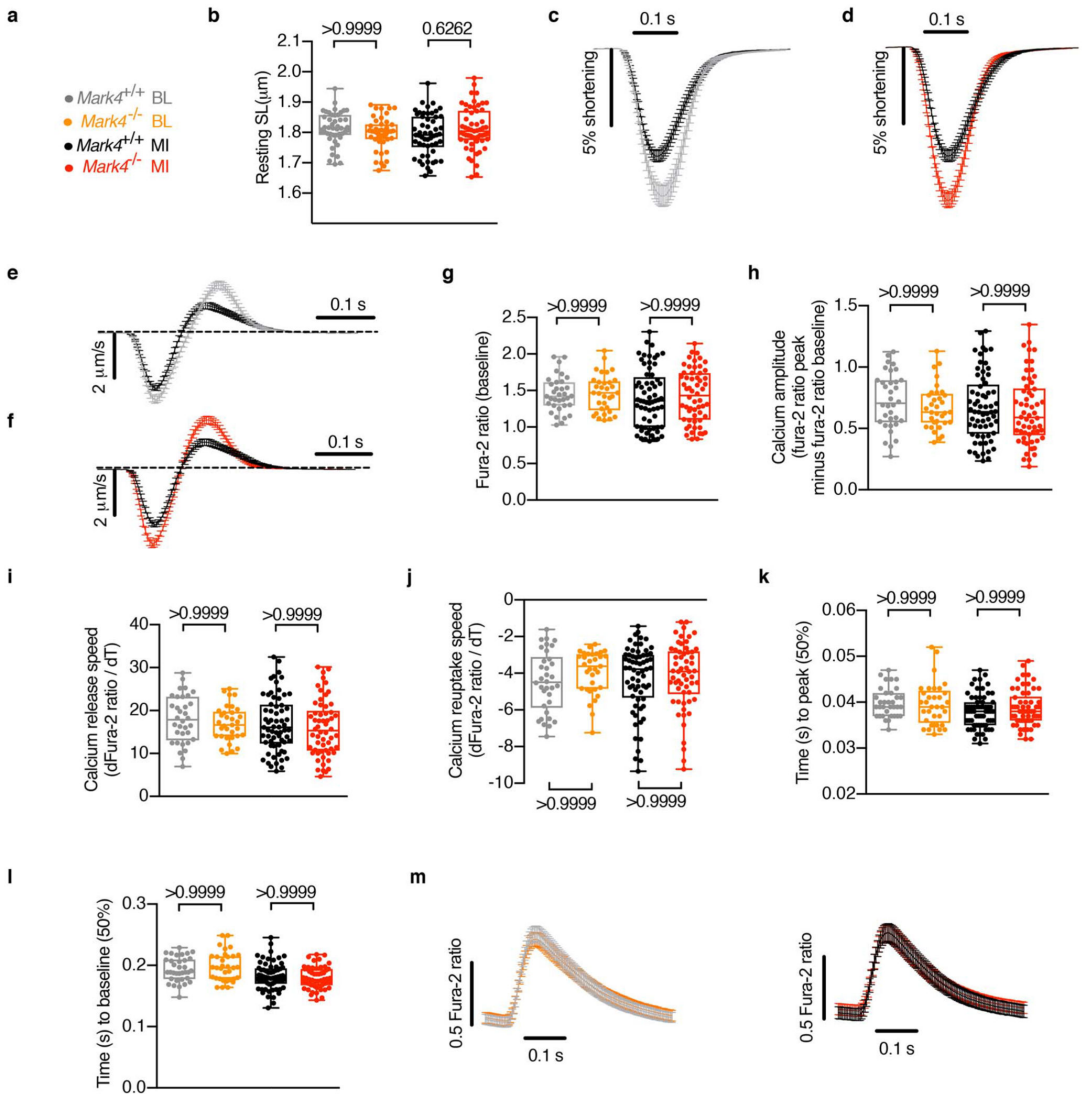
The effect of MARK4 deficiency on sarcomere length, peak shortening, velocity, and calcium transients in cardiomyocytes before and after myocardial infarction. **a-f**, Contractility assay of single primary cardiomyocytes (CMs) isolated at baseline (BL) or at day 3 post-myocardial infarction (MI) from the following groups: *Mark4* ^+/+^ BL (n=4 mice / n=45 CMs examined over 4 independent experiments), *Mark4* ^-/-^ BL (n=3 mice / n=45 CMs examined over 3 independent experiments), *Mark4* ^+/+^ MI (n=5 mice / n=54 CMs examined over 5 independent experiments), and *Mark4* ^-/-^ MI (n=6 independent mice / n=57 CMs examined over 6 independent experiments). Colour denotation of samples (**a**). Resting sarcomere length (SL) (**b**). Average sarcomere shortening traces were compared (**c-d**). Average velocity traces (dSL/dT) (**e-f**). **g-m**, Calcium influx assay on single CMs isolated from *Mark4* ^-/-^ or control mice at baseline or at day 3 post- MI in the following groups: *Mark4* ^+/+^ BL group (n=2 mice / n=34 CMs examined over 2 independent experiments), *Mark4* ^-/-^ BL groups (n=2 mice / n=33 CMs examined over 2 independent experiments), *Mark4* ^+/+^ MI group (n=4 mice / n=65 CMs examined over 4 independent experiments), *Mark4* ^-/-^ MI groups (n=3 mice / n=58 CMs examined over 3 independent experiments). Basal Ca^2+^ level (**g**). Amplitude level of Ca^2+^ transient (**h**). Ca^2+^ release speed during contraction (**i**). Ca^2+^ reuptake speed during contraction (**j**). Ca^2+^ elevation time (**k**). Ca^2+^ reuptake time (**l**). Traces of Ca^2+^ kinetic curves (**m**). The box bounds represent the 25^th^ and 75^th^ percentiles, the middle line shows the median, and the whiskers show the minimum and maximum (**b, g-l**). Mean ± s.e.m.; two-way ANOVA with Bonferroni post-hoc correction for multiple comparisons (**b, g-l**). *P* values are indicated on the graphs.

To examine the effect of MARK4 on cardiomyocyte function, we subjected freshly isolated primary cardiomyocytes^28^ from wild-type and *Mark4* ^-/-^ mice to a single cell contractility assay using an electrical stimulator (Fig.2f-2j). We found that sarcomere peak shortening of isolated cardiomyocytes strongly correlated with the *in vivo* LVEF (Fig.2g), indicating that isolated cardiomyocyte contraction measured *ex vivo* reflects LVEF assessed *in vivo* (Fig.1d, Fig.2a, and Fig.2e). At baseline (BL), wild-type and MARK4-deficient cardiomyocytes had similar levels of resting sarcomere length (Extended Data Fig.4a-4b), sarcomere peak shortening and contraction/relaxation velocities (Fig.2h-2j), an observation consistent with the absence of LVEF difference between wild-type and *Mark4* ^-/-^ mice prior to MI (Fig.1d). After MI, wild-type cardiomyocytes displayed markedly reduced sarcomere shortening (lower by 22.5% ±3.7%) (Fig.2h; Extended Data Fig.4c), with slower relaxation velocity (lower by 25.2%±4.4%) (Fig.2j; Extended Data Fig.4e), when compared with cardiomyocytes isolated from wild-type mice without MI. Strikingly, although no difference in resting sarcomere length was observed between *Mark4* ^-/-^ and wild-type cardiomyocytes after MI (Extended Data Fig.4b), *Mark4* ^-/-^ cardiomyocytes displayed a greater level of sarcomere shortening (higher by 36.0%±6.0%) (Fig.2h; Extended Data Fig.4d) together with a greater velocity during both the contraction (higher by 42.0%±6.9%) and relaxation (higher by 46.7%±7.5%) phases (Fig.2i-2j; Extended Data Fig.4f) when compared with wild-type cells. Upstream changes of calcium influx in excitation-contraction coupling could contribute to the contractile alterations, however, we did not observe any significant difference of Ca^2+^ transients between the electrically stimulated *Mark4* ^-/-^ and wild-type cardiomyocytes at baseline or at day 3 post-MI (Extended Data Fig.4g-4m). These data strongly demonstrate that MARK4 deficiency substantially improves both contractile and relaxation functions of cardiomyocytes after MI.

## MARK4 alters microtubule detyrosination

**Fig. 3:**
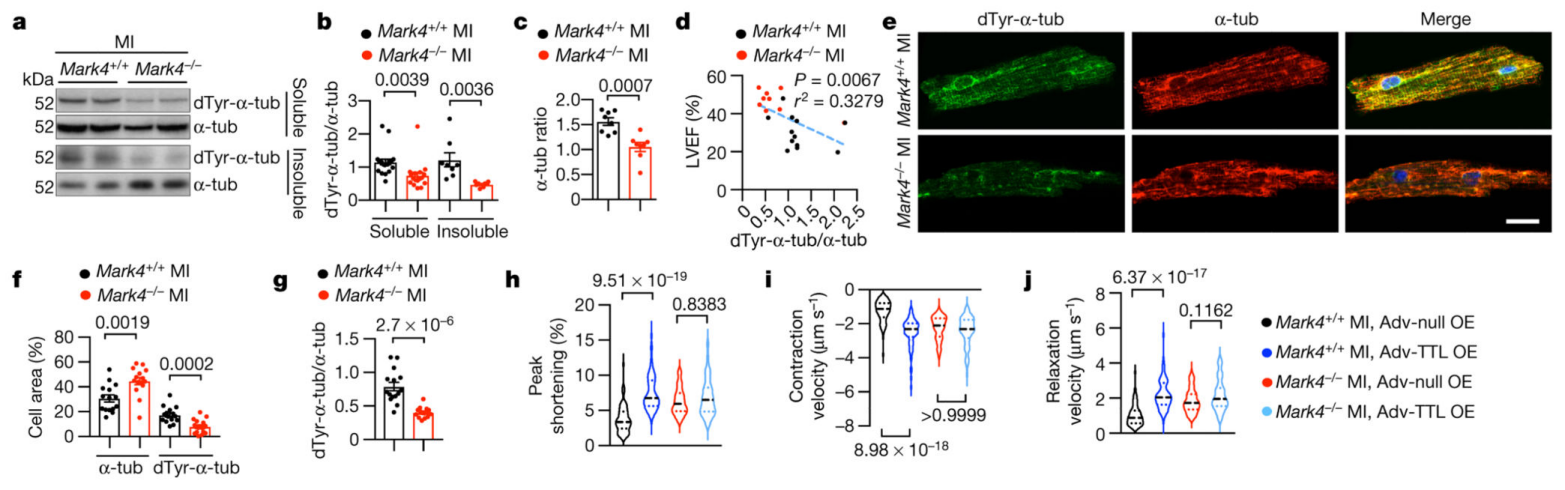
MARK4 regulates cardiomyocyte contractility by promoting microtubule detyrosination. **a-d**, Western blots (WBs) of whole heart extraction from mice at day 3 post-myocardial infarction (MI), in soluble (S.) and insoluble (Ins.) fractions. dTyr-tub: detyrosinated α-tubulin. α-tub: α-tubulin. Representative WBs (**a**)**.** Ratio of dTyr-tubulin over total α-tubulin in the following groups: *Mark4* ^+/+^ MI soluble (n=20), *Mark4* ^-/-^ MI soluble (n=17), *Mark4* ^+/+^ MI insoluble (n=8), and *Mark4* ^-/-^ MI insoluble (n=8) (**b**). Ratio of α-tub in the soluble fraction over α-tub in the insoluble fraction (n=8 mice per group) (**c**). Correlation between left ventricular ejection fraction (LVEF) and ratio of dTyr-tubulin/α-tub, in *Mark4* ^-/-^ (n=9) and control mice (n=12) (**d**). **e-g,** Confocal images of the isolated cardiomyocytes (CMs) at day 3 post-MI. Representative images, scale bar= 20 μm (**e**). Percentage (%) of dTyr-tub or total α-tub area per cell (**f**), and ratio of dTyr-tub/total α-tub (n=3 mice / n=15 CMs per group) (**g**). **h-q,** Adenovirus (Adv)-mediated overexpression (o.e.) of TTL in primary cardiomyocytes isolated from *Mark4* ^-/-^ or control mice at day 3 post-MI, with o.e. of a null as controls (Ctrl). Contractility assay of single CMs in the following groups: *Mark4* ^+/+^ MI Adv-Null (n=3 mice / n=75 CMs examined over 3 independent experiments), *Mark4* ^+/+^ MI Adv-TTL (n=3 mice / n=69 CMs examined over 3 independent experiments), *Mark4* ^-/-^ MI Adv-Null (n=3 mice / n=74 CMs examined over 3 independent experiments), and *Mark4* ^-/-^ MI Adv-TTL (n=3 mice / n= 73 CMs examined over 3 independent experiments). Colour denotation of samples (**h**). Sarcomere peak shortening (**i**). Pooled data of contraction (**j**) and relaxation (**k**) velocity. Violin plots lines at the median and quartiles (**i-k**). Mean ± s.e.m.; two-tailed unpaired *t*-test (**b, c, f, g**); two-tailed correlation test (**d**); two-way ANOVA with Bonferroni post-hoc correction for multiple comparisons (**i, j, k**). *P* values are indicated on the graphs.

Detyrosinated MTs represent tunable, compression-resistant elements that impair cardiac function in the human failing hearts^5,29^. We confirmed that detyrosinated α-tubulin level was significantly higher in cardiomyocytes isolated from ischaemic hearts compared with cardiomyocytes isolated from sham animals, in contrast with the remaining cell pool (immune cells, fibroblast, endothelial cells) which did not display such a change in α-tubulin detyrosination (Extended Data Fig.2d-2e). Previous data indicate that MARK4 affects posttranslational microtubule detyrosination and polyglutamylation in ciliated cells^30^. Therefore, we hypothesized that MARK4 deficiency may affect microtubule detyrosination in cardiomyocytes after MI. We found a significantly lower level of detyrosinated microtubules in whole heart tissue extracts (Fig.3a-3b), and in isolated cardiomyocytes (together with reduced polyglutamylated microtubules) (Fig.3e-3g; Extended Data Fig.2f-2g) of *Mark4* ^-/-^ mice compared with their littermate wild-type controls after MI. In the absence of MARK4, we observed reduced ratio of α-tubulin in the soluble fraction versus its level in the insoluble fraction (Fig.3c), indicating a reduced percentage of free tubulin level without MARK4. More interestingly, we found that the level of tubulin detyrosination inversely correlated with LVEF (Fig.3d), suggesting a major role of MARK4-dependent modulation of microtubule detyrosination in controlling cardiac function after MI.

**Extended Data Fig. 5:**
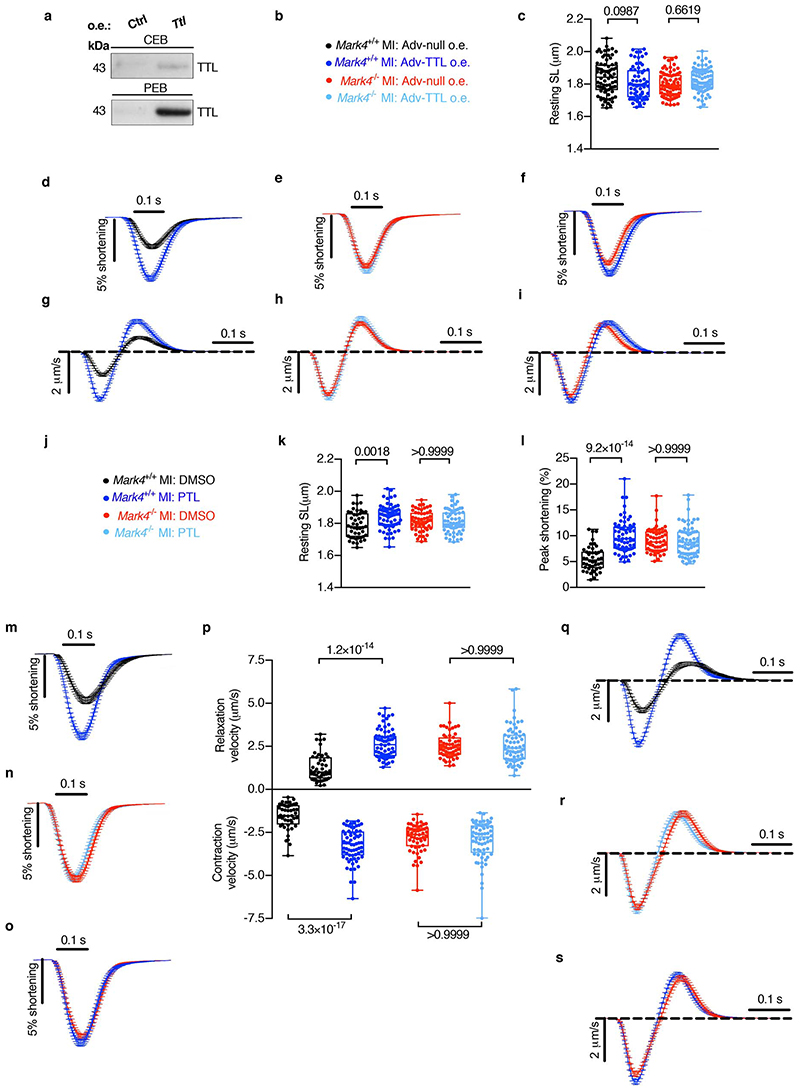
The effect of TTL overexpression, or PTL treatment, on contractility of *Mark4* ^-/-^ cardiomyocytes after myocardial infarction. **a-i**, Adenovirus (Adv)-mediated overexpression (o.e.) of Tubulin Tyrosine Ligase (TTL) in cardiomyocytes isolated from *Mark4* ^-/-^ or control *Mark4* ^+/+^ mice at day 3 post-myocardial infarction (MI), with o.e. of a null as control (Ctrl). Representative western blot (**a**). Contractility assay of single CMs with o.e. in the following groups: *Mark4* ^+/+^ MI Adv-Null (n=3 mice/n=75 CMs examined over 3 independent experiments), *Mark4* ^+/+^ MI Adv-TTL (n=3 mice / n=69 CMs examined over 3 independent experiments), *Mark4* ^-/-^ MI Adv-Null (n=3 mice / n=74 CMs examined over 3 independent experiments), and *Mark4* ^-/-^ MI Adv-TTL (n=3 mice / n= 73 CMs examined over 3 independent experiments). Colour denotation of samples (**b**). Resting sarcomere length (SL) (**c**). Average sarcomere shortening traces (**d-f**). Average velocity traces (dSL/dT) (**g-i**). **j-s**, Contractility assay of single CMs isolated at day 3 post-MI with the following treatments: *Mark4* ^+/+^ MI DMSO (n=3 mice / n=46 CMs examined over 3 independent experiments), *Mark4* ^+/+^ MI PTL ( n=3 mice / n=67 CMs examined over 3 independent experiments), *Mark4* ^-/-^ MI DMSO (n=3 mice / n=55 CMs examined over 3 independent experiments), and *Mark4* ^-/-^ MI PTL (n=3 mice / n=64 CMs examined over 3 independent experiments). Color denotation of samples (**j**). Resting sarcomere length (**k**). Sarcomere peak shortening (**l**). Average sarcomere shortening traces (**m-o**). Pooled data of contraction velocity and relaxation velocity (**p**). Average velocity traces (dSL/dT) (**q-s**). The box bounds represent the 25^th^ and 75^th^ percentiles, the middle line shows the median, and the whiskers show the minimum and maximum (**c, k, l, p**). Mean±s.e.m.; two-way ANOVA test with Bonferroni post-hoc correction for multiple comparisons (**c, k, l, p**). *P* values are indicated on the graphs.

To further address the hypothesis that MARK4 deficiency improves cardiomyocyte contractility through its impact on microtubule detyrosination, we employed a genetic approach to overexpress tubulin tyrosine ligase (TTL) using an adenovirus system (Extended Data Fig.5a-5c) to reverse the effect of TCP^31^ (Fig.3h-3k). TTL overexpression robustly improved peak shortening (Fig.3i; Extended Data Fig.5d) and increased the velocity of both contraction and relaxation (Fig.3j-3k; Extended Data Fig.5g) of failing wild-type cardiomycytes^7^. However, overexpression of TTL could not further improve peak shortening (Fig. 3i; Extended Data Fig.5e) and contractile velocities of post-MI *Mark4* ^-/-^ cardiomyocytes (Fig. 3j-3k; Extended Data Fig.5h), consistent with the already low level of detyrosinated microtubules in *Mark*4^-/-^ cardiomyocytes. We further confirmed these data by using a pharmacological approach with parthenolide (PTL) to inhibit microtubule detyrosination^5,7^(Extended Data Fig.5j-5s). Taken together, our data show that MARK4 regulates cardiac inotropic function through its impact on microtubule detyrosination in cardiomyocytes.

## MARK4 directs VASH2 access to MTs

**Extended Data Fig. 6:**
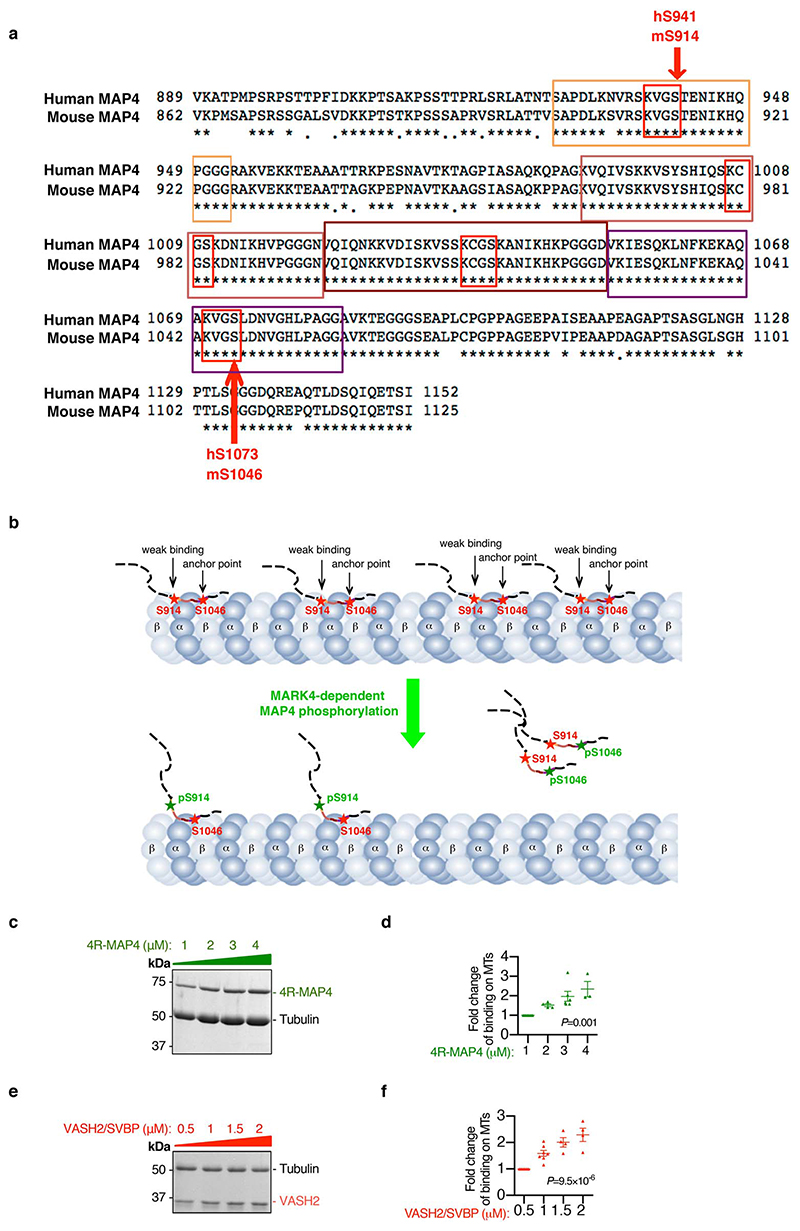
The association of MAP4 or VASH2 with the polymerized microtubules. **a,** Protein sequence alignment between human MAP4 (NP002366) and mouse MAP4 (NP001192259). KXGS motifs (highlighted with red frames) within the tubulin binding repeats (highlighted with yellow, brown, dark brown, and purple frame) of MAP4 are MARK4 substrate sites. S941 of human MAP4 (S914 of mouse MAP4) and S1073 of human MAP4 (S1046 of mouse MAP4) are conserved phosphorylation sites within KXGS motifs. **b**, Schematic illustration of possible association between MAP4 and microtubules pre- or post- MARK4-dependent phosphorylation. Non-phosphorylated MAP4 binds to microtubules. Upon MARK4-dependent phosphorylation of mS914 at the microtubule weak binding site, MAP4 makes allosteric changes. Upon MARK4-dependent phosphorylation of mS1046 at the microtubule anchor site, MAP4 detaches from microtubules. **c-d**, Representative gel image of 4R-MAP4 (1-4 μM) binding to the polymerized microtubules (MTs) (5 μM) in a microtubule co-sedimentation assay (**c**). Quantification of the binding (**d**). n=7 samples examined over 3 independent experiments (1 μM); n=4 samples examined over 3 independent experiments (2 μM); n=6 samples examined over 3 independent experiments (3 μM); n=3 samples examined over 3 independent experiments (4 μM). **e-f**, Representative gel image of VASH2/SVBP (0.5-2 μM) binding to the polymerized MTs (2.5 μM) in a microtubule co-sedimentation assay (**e**). Quantification of the binding (**f**). n=7 samples examined over 5 independent experiments (0.5 μM); n=7 samples examined over 5 independent experiments (1 μM); n=4 samples examined over 3 independent experiments (1.5 μM); n=4 samples examined over 3 independent experiments (2 μM). Mean±s.e.m.; one-way ANOVA test (**d, f**). *P* values are indicated on the graphs.

**Fig. 4:**
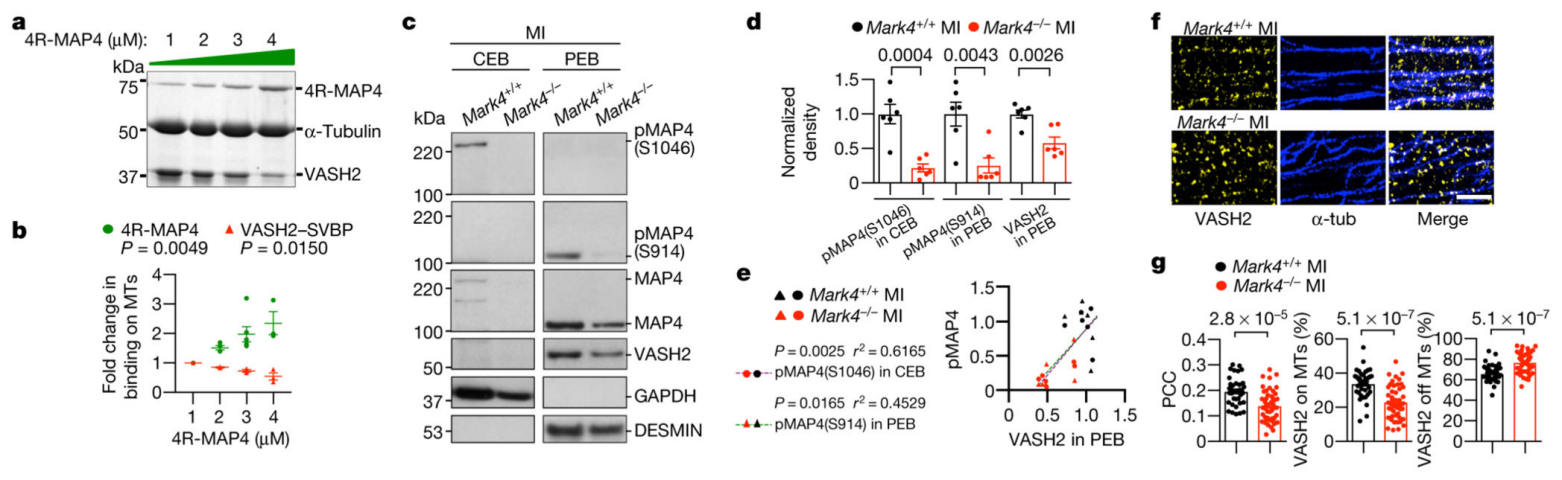
MARK4 controls microtubule detyrosination through MAP4 phosphorylation to facilitate VASH2 access to microtubules. **a-b**, Representative gel image of VASH2/SVBP (3 μM) binding to polymerized microtubules (MTs) (5 μM) in the presence of different amounts of 4R-MAP4 (1-4 μM) in a microtubule co-sedimentation assay (**a**). Quantification of the binding (**b**). n=3 independent experiments per group. **c-e**, Subcellular fractionations on *Mark4* ^-/-^ or control cardiomyocytes (CMs) isolated post-myocardial infarction (MI). Representative western blots of the fractions from cytosolic extraction buffer (CEB) or pellet extraction buffer (PEB) derived from the same experiment (**c**). Quantification of pMAP4^S1046^ in CEB, pMAP4^S914^ in PEB, and VASH2 level in PEB (n=6 mice per group, blots were processed in parallel) (**d**). Correlation between VASH2 level in the PEB fraction and phosphorylated MAP4 (pMAP4) levels (**e**). **f-g**, STED images of VASH2 and α-tubulin (α-tub) in *Mark4* ^-/-^ or control CMs isolated from mice post-MI. Representative images, scale bar=2 μm (**f**). Pearson Correlation Coefficient (PCC) of VASH2 and α-tubulin signals, percentage (%) of VASH2 signals on the polymerized MTs, and percentage of VASH2 signals off the polymerized MTs, in *Mark4* ^+/+^ MI group (n=6 mice / n=38 CMs examined over 3 independent experiments), and data of *Mark4* ^-/-^ MI group (n=6 mice / n=47 CMs examined over 3 independent experiments) (**g**). Mean ± s.e.m.; one-way ANOVA test (**b**); two-tailed unpaired *t*-test (**d, g**); two-tailed correlation test (**e**). *P* values are indicated on the graphs.

Detyrosination of α-tubulin preferentially occurs on polymerized microtubules^32^. Apart from binding to VASH, C-terminal tubulin tails of the polymerized microtubules are also important for MAP binding^33,34^. MAP4 bound to the C-terminal tubulin tail along the protofilament stabilizes the longitudinal contacts of the microtubule, and this interaction can affect other microtubule binding partners such as the motor protein Kinesin-1^34^. MARK4, as a kinase, is expected to phosphorylate MAP4 on its KXGS motif (including S941 and S1073 in human MAP4, or S914 and S1046 in murine MAP4) within its microtubule-binding repeats^19,20^ (Extended Data Fig.6a), and alters MAP4 binding status on the protofilament (Extended Data Fig.6b). We therefore hypothesized that MARK4, through modifying MAP4 phosphorylation, may affect VASH accessibility to C-terminal α-tubulin tail and therefore influence microtubule detyrosination. To address this, we firstly used an *in vitro* microtubule co-sedimentation assay. Both MAP4 (Extended Data Fig.6c-6d) and VASH2/SVBP (Extended Data Fig.6e-6f) were able to incrementally bind to polymerized microtubules when incremental amounts were separately applied in the assays, consistent with the results of the past studies^12,34^. Interestingly, we found that VASH2/SVBP bound to polymerized microtubules gradually decreased in the presence of incremental amounts of previously bound MAP4 (with four microtubule-binding repeats, 4R-MAP4) (Fig.4a-4b). Therefore, these results support the hypothesis that the level of MAP4 occupancy on the polymerized microtubules influences the level of VASH2 access to the microtubule protofilaments.

**Extended Data Fig. 7:**
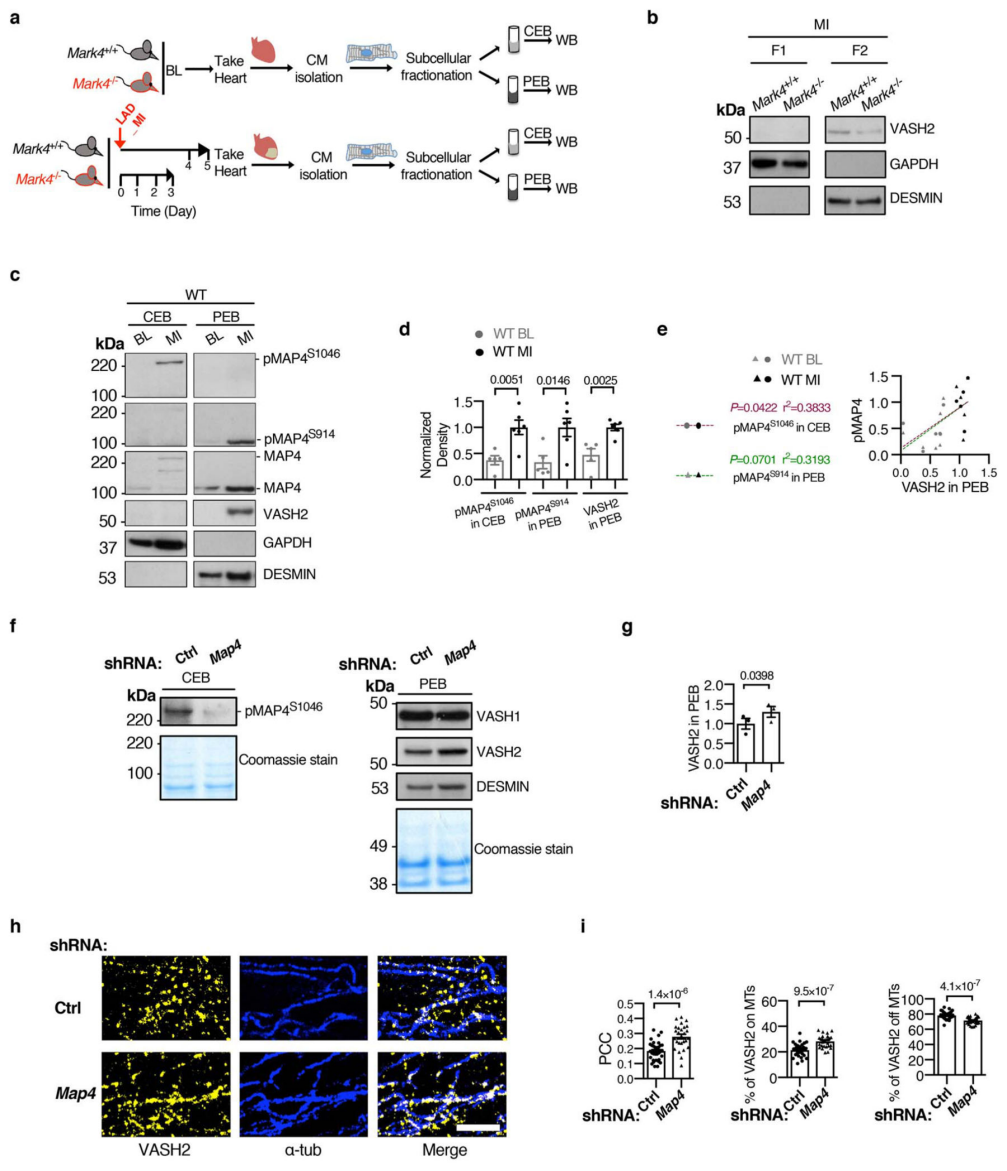
Association of VASH2 with cardiomyocyte microtubules pre- and post-myocardial infarction, and impact of MAP4 knock-down. **a,** Subcellular fractionation on primary cardiomyocytes (CMs) isolated from mice at baseline (BL) or post-myocardial infarction (MI). Western blotting (WB) of the fractions from cytosolic extraction buffer (CEB) or pellet extraction buffer (PEB). **b**, Representative WBs of F1 (free tubulin fraction) and F2 (extraction from the stable pellet fraction) fractions obtained from a conventional fractionation method. **c-e**, WBs of CEB or PEB fractions of wild-type (WT) CMs at baseline, or post-MI. Representative WBs (derived from the same experiment) (**c**). Quantification of pMAP4^S1046^ in CEB, pMAP4^S914^ in PEB, and VASH2 levels in PEB (n=5 mice at BL, n=6 mice post-MI, blots were processed in parallel) (**d**). Correlation between VASH2 level in the PEB fraction and phosphorylated MAP4 (pMAP4) levels (**e**). **f-i**, WT post-MI CMs transduced with adenovirus (Adv)-mediated shRNA *Map4*, or control (Ctrl). Representative WBs of CEB fraction or PEB fraction, and Coomassie stained gels loaded with the same amounts of proteins (**f**). Quantification of VASH2 levels in PEB (n=3 mice examined over 3 experiments per group) (**g**). (**h-i**), STED images of VASH2 and α-tubulin (α-tub) in the cardiomyocytes after knocking down MAP4. Representative images, scale bar=2 μm (**h**). Pearson Correlation Coefficient (PCC) of VASH2 and α-tubulin (α-tub) signals. percentage (%) of VASH2 signals on the polymerized microtubules (MTs), and percentage of VASH2 signals off the polymerized MTs, in the following groups (**i**): shRNA Ctrl (n=2 mice / n=35 CMs examined over 2 independent experiments), and shRNA *Map4* (n=2 mice / n=27 CMs examined over 2 independent experiments ). Mean±s.e.m.; two-tailed unpaired *t*-test (**d, g, i**); two-tailed correlation test (**e**). *P* values are indicated on the graphs.

**Extended Data Fig. 8:**
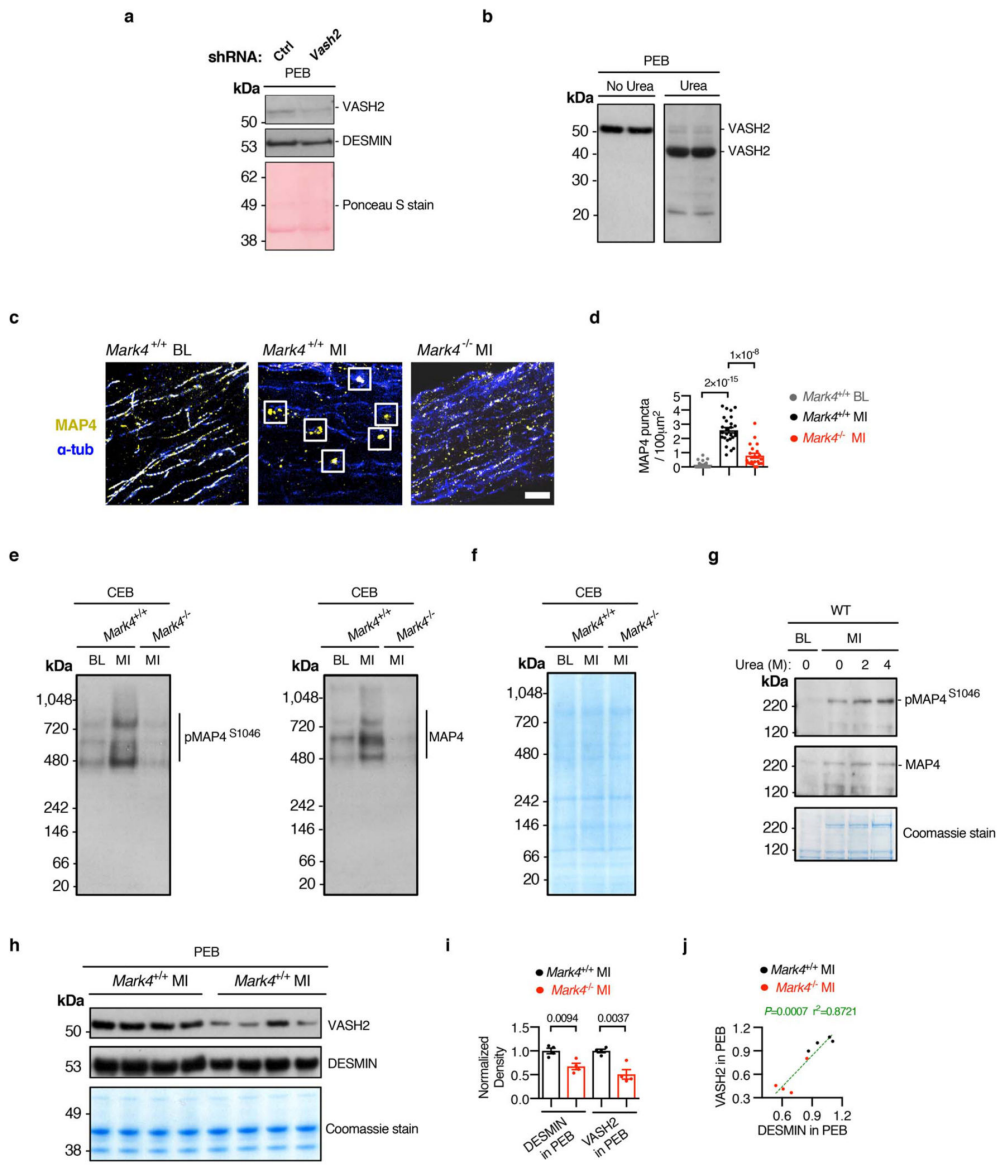
The status of VASH2 and MAP4 in cardiomyocytes pre- and post-myocardial infarction. **a**, Subcellular fractionation on wild-type (WT) cardiomyocytes (CMs), isolated from mice post-myocardial infarction (MI) and transduced with adenovirus-mediated shRNA *Vash2* or control (Ctrl). Representative western blots (WBs) of fraction in pellet extraction buffer (PEB), with the same membrane stained with Ponceau S. **b**, Representative WB of PEB extractions denatured in the presence of urea (or not), from post-MI CMs. **c-d**, STED images of MAP4 and α-tubulin (α-tub) in CMs of *Mark4* ^-/-^ or control mice at baseline (BL) or post-MI. Oligomerized puncta are indicated within the square frames. Representative images, scale bar=2 μm (**c**). Quantification of the presence of the MAP4 oligomerized puncta in the following groups (**d**): *Mark4* ^+/+^ BL (n= 2 mice / n= 22 CMs examined over 2 independent experiments), *Mark4* ^+/+^ MI (n=2 mice / n=26 CMs examined over 2 independent experiments), and *Mark4* ^-/-^ MI (n=2 mice / n=21 CMs examined over 2 independent experiments). **e-g**, WB of native gels loaded with samples in cytosolic extraction buffer (CEB) of CMs isolated at baseline (BL) or post-MI. The presence of pMAP4^S1046^ and total MAP4 is indicated (**e**). Coomassie stained native gel loaded with the same amounts of proteins as used in e (**f**). WB of CEB fraction denatured in the presence of urea, with Coomassie stained denaturing gel loaded with the same amounts of protein (**g**). **h-j**, WB of fractions in PEB, of CMs isolated from *Mark4* ^-/-^ or control mice post-MI, with Coomassie stained gel loaded with the same amounts of proteins (**h**). Quantification of VASH2 and DESMIN levels in PEB fraction (n= 4 mice per group) (**i**). Correlation between DESMIN and VASH2 levels in PEB (**j**). Mean±s.e.m.; two-tailed unpaired *t*-test (**d, i**); two-tailed correlation test (**j**). *P* values are indicated on the graphs.

To confirm this hypothesis in *in vivo*, we performed biochemical subcellular fractionation on primary cardiomyocytes isolated from non-ischaemic and ischaemic hearts of wild-type and *Mark4* ^-/-^ mice using a commercial kit, which we have validated (Extended Data Fig.7a-7b). We firstly confirmed that MAP4 was expressed in the cardiomyocytes and its level was higher post-MI (Extended Data Fig.7c), a result consistent with data showing that MAP4 levels significantly increase in human hearts with cardiomyopathies^7^. MAP4 was detected in its S914 phosphorylated (within KXGS motif) form (pMAP4^S914^) in the pellet extraction buffer (PEB), and also in its S1046 form (pMAP4^S1046^) in the cytosolic extraction buffer (CEB) (Extended Data Fig.7c-7e). Knocking down MAP4 by small hairpin RNA (shRNA) in the isolated cardiomyocytes post-MI led to increased VASH2 levels in the PEB fraction, confirmed by both western blot and immunocytochemistry (Extended Data Fig.7f-7i), which was in line with the results of *in vitro* microtubule co-sedimentation assay (Fig.4a-4b). VASH2 was detected as a specific band (validated by specific knock-down using shRNA, Extended Data Fig.8a) of around 50 kDa in the PEB fraction (Extended Data Fig.8a-8b), higher than its theoretical molecular weight of 40 kDa, presumably due to the formation of a stable complex with SVBP, because adding a denaturing agent (urea) reduced its size to around 40 kDa (Extended Data Fig.8b). Upon MI, pMAP4^S914^ was detected as a 110 kDa form in the PEB fraction whereas pMAP4^S1046^ was detected as a 220 kDa form in the CEB fraction (Fig.4c and Extended Data Fig.7c). MAP4 was detected as giant puncta in the cytosol of cardiomyocytes isolated post-MI, and these puncta were barely present at baseline (Extended Data Fig.8c-8d). pMAP4^S1046^ (in the CEB fraction) formed oligomerized structures (at 440 kDa or higher) as revealed on the native gel (Extended Data Fig.8e-8f), and these pMAP4^S1046^ oligomers could be further reduced to the 220 kDa form in presence of urea as revealed on the denaturing gel (Extended Data Fig.8g). The data suggest that MAP4 phosphorylation at S1046 is associated with its presence as oligomers/giant puncta in the cytosol *in situ*. Our results are consistent with a structural model, in which S914 is within the weak microtubule binding repeat of MAP4, whereas S1046 is within the strong anchor point of MAP4 binding repeat to the microtubules^34^ (Extended Data Fig.6b), so that S1046 phosphorylation can lead to detachment of MAP4 from polymerized microtubules and accumulation in the cytosol. Accordingly, a higher pMAP4^S1046^ level was strongly and positively correlated with increased VASH2 levels in the PEB fraction (there also was a weaker correlation between pMAP4^S914^ levels and VASH2 levels in the PEB fraction) (Extended Data Fig.7c, 7e) in wild-type cardiomyocytes, indicating an association between phosphorylated MAP4 (at S941 and S1046) levels and VASH2 levels on the polymerized microtubules. Strikingly, levels of pMAP4^S914^ and pMAP4^S1046^ were substantially reduced in *Mark4* ^-/-^ cardiomyocytes after MI (Fig.4c-4d), confirming S914 and S1046 of MAP4 as MARK4 kinase substrate sites. Reduced levels of pMAP4^S1046^ in the CEB fraction and pMAP4^S914^ in the PEB fraction correlated well with a reduced level of VASH2 in the PEB fraction (r^2^=0.6165, *P*=0.0025; r^2^=0.4529, *P*=0.0165, respectively) (Fig.4c-4e), with a stronger association between pMAP4^S1046^ and VASH2. In addition, we found that VASH2 levels were positively correlated with DESMIN levels in the PEB fraction (Extended Data Fig.8h-8j), supporting previous data that detyrosinated microtubules are positively correlated with DESMIN levels in cardiomyocytes^5^. In summary, our results suggest that MARK4 kinase, through phosphorylation of MAP4 at S914 and S1046, changes MAP4 status to allow VASH2 access to the polymerized microtubule for its TCP activity.

**Extended Data Fig. 9:**
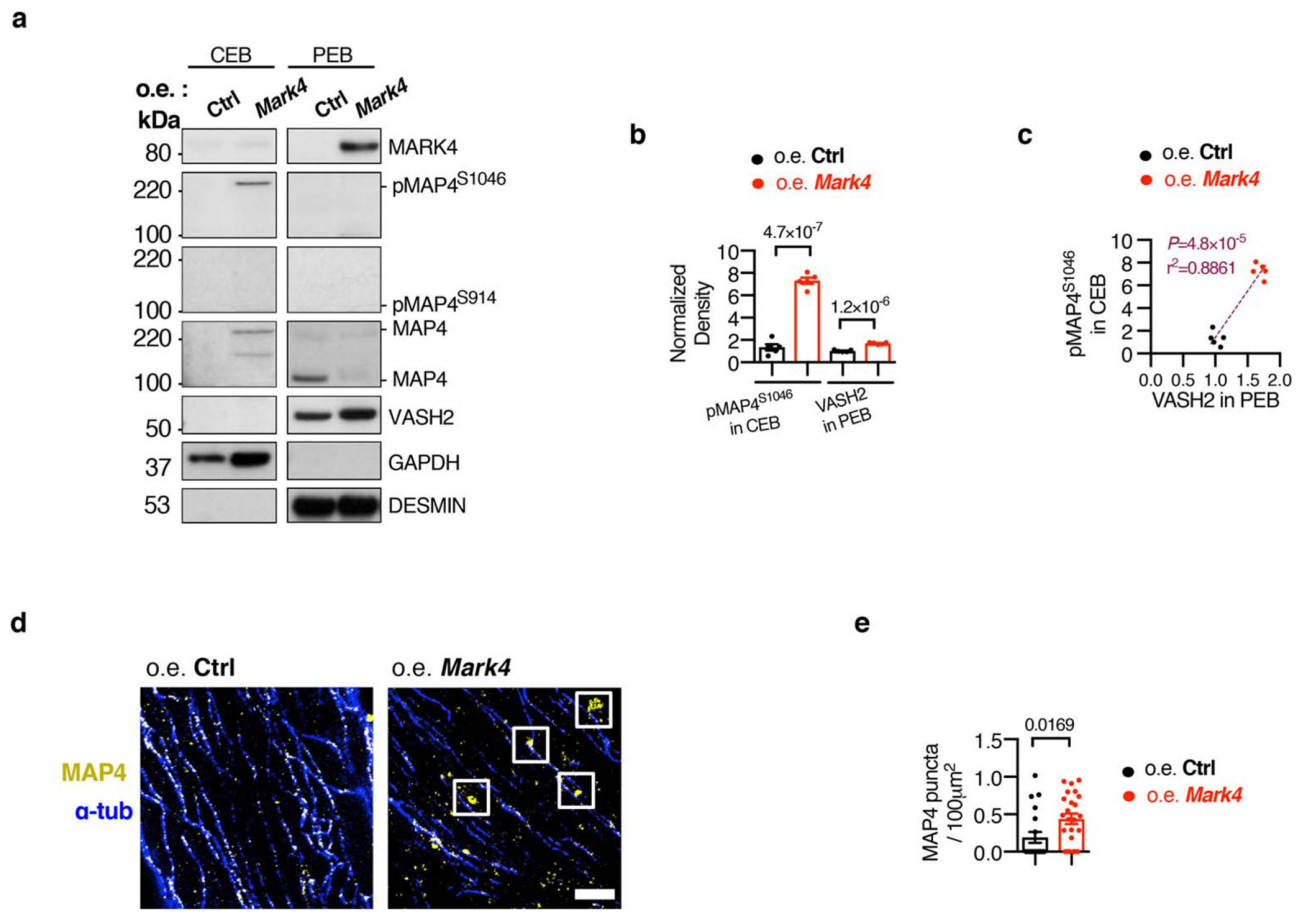
MARK4 overexpression regulates MAP4 phosphorylation, and presence of MAP4 oligomers in the cytosolic fraction. **a-c**, Subcellular fractionation on wild-type cardiomyocytes (CMs) transduced with adenovirus to overexpress (o.e.) *Mark4* or a null control (Ctrl). Representative western blots (WBs) of fractions in cytosolic extraction buffer (CEB) or pellet extraction buffer (PEB) (derived from the same experiment) (**a**). Quantification of pMAP4^S1046^ in CEB, and VASH2 level in PEB (n=5 mice per group, blots were processed in parallel) (**b**). Correlation between VASH2 level in the PEB fraction and phosphorylated MAP4 (pMAP4) levels (**c**). **d-e**, STED images of MAP4 and α-tubulin (α-tub) in wild-type baseline CMs transduced with adenovirus to overexpress *Mark4* or a null control. Representative images, scale bar=2 μm (**d**). Quantification of MAP4 oligomerized puncta in the following groups (**e**): o.e. Ctrl (n=2 mice / n=20 CMs examined over 2 independent experiments), and o.e. *Mark4* (n= 2 mice / n= 24 CMs examined over 2 independent experiments). Mean ± s.e.m.; two-tailed unpaired *t*-test (**d, e**); two-tailed correlation test (**c**). *P* values are indicated on the graphs.

**Extended Data Fig. 10:**
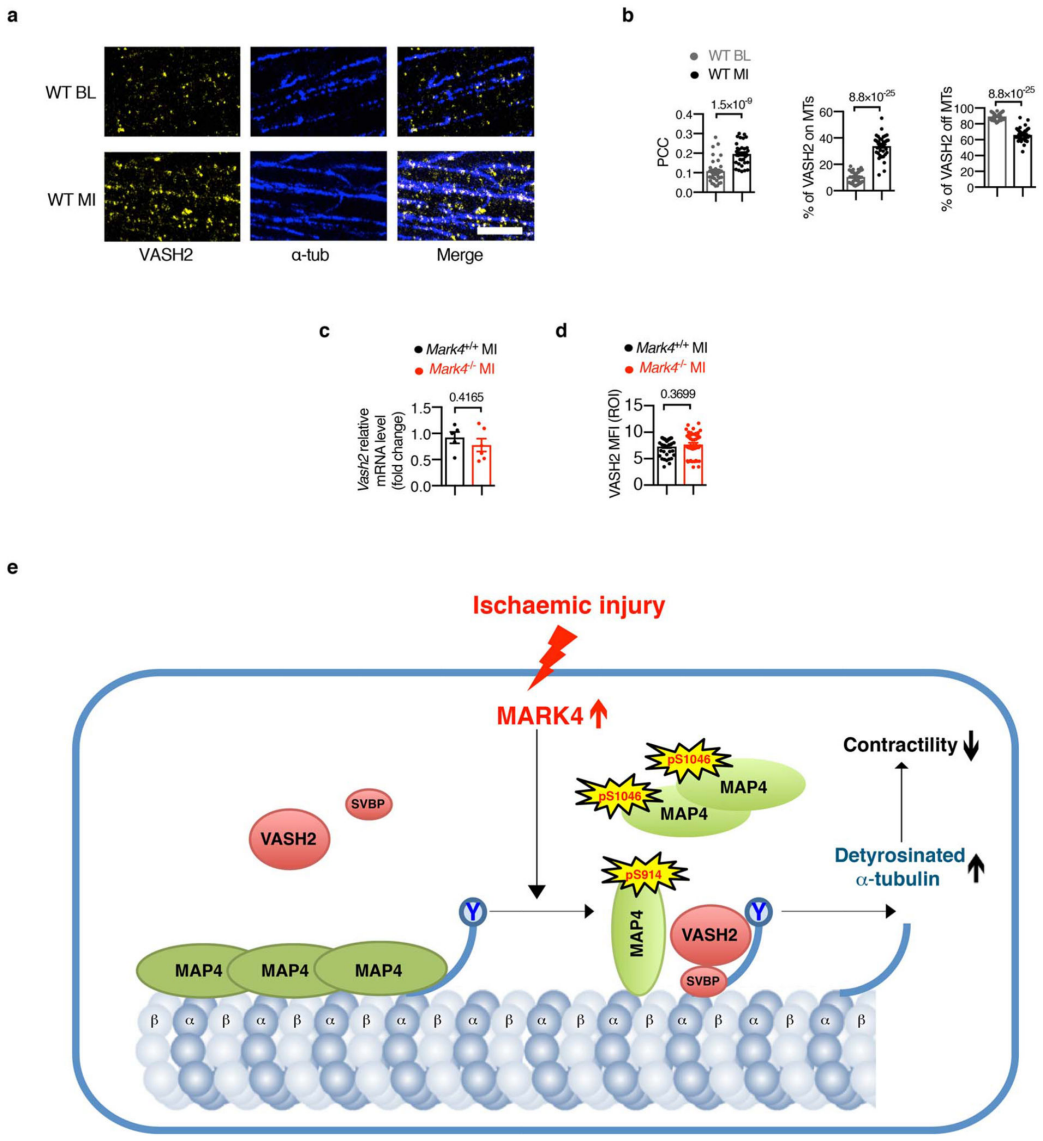
VASH2 status in cardiomyocytes pre- and post-myocardial infarction, and the schematic summary of the results. **a-b**, STED images of VASH2 and α-tubulin (α-tub) in wild-type (WT) cardiomyocytes (CMs) at baseline (BL) or post-myocardial infarction (MI). Representative images, scale bar=2 μm (**a**). Pearson Correlation Coefficient (PCC) of VASH2 and α-tub signals, percentage (%) of VASH2 signals on the polymerized microtubules (MTs), and percentage of VASH2 signals off the MTs, in the following groups (**b**): WT BL (n=4 mice / n=38 CMs examined over 2 independent experiments), and WT MI (n=38 CMs of n=6 mice / n=38 CMs examined over 3 independent experiments). **c,** Real-time PCR on post-MI CMs, from the following groups: *Mark4* ^+/+^ MI (n= 5 mice), and *Mark4* ^-/-^ MI (n=6 mice). **d**, Quantification of VASH2 mean fluorescence intensity (MFI) within cell area (region of interest, ROI) using the STED images from the following groups: *Mark4* ^+/+^ MI (n=6 mice / n= 38 CMs examined over 3 independent experiments), and *Mark4* ^-/-^ MI (n= 6 mice/ n= 47 CMs examined over 3 independent experiments). Mean ± s.e.m.; two-tailed unpaired *t*-test (**b, c, d**). *P* values are indicated on the graphs. **e,** A working model for MARK4-dependent regulation of microtubule detyrosination after MI: Upon ischaemic injury, increased MARK4 phosphorylates MAP4 at its KXGS motifs. Phosphorylated MAP4 either changes its conformation on the polymerized microtubules, or detaches itself from the polymerized microtubules to form oligomerized MAP4 structures in the cytosol. The phosphorylation of MAP4 by MARK4 allows for space access of VASH2 to the polymerized microtubules, thereby promoting α-tubulin detyrosination. As a consequence, the increased level of detyrosinated microtubules causes a reduction in contractile function of the cardiomyocyte.

To further confirm the causal effect of MARK4 on VASH2 localization, we overexpressed MARK4 in primary cardiomyocytes, which caused the appearance of pMAP4^S1046^ (Extended Data Fig.9a-9c) and giant MAP4 puncta in the cytosol (Extended Data Fig.9d-9e), and led to increased VASH2 levels in the PEB fraction (Extended Data Fig.9a-9c). By using stimulated emission depletion (STED) super-resolution microscopy^35^, we found a strong co-localization of VASH2 on the polymerized microtubules in primary cardiomyocytes isolated from wild-type hearts post-MI when compared with the samples isolated from wild-type at baseline (Extended Data Fig.10a-10b). Total VASH2 levels were comparable between *Mark4* ^-/-^ cardiomyocytes and *Mark4* ^+/+^ cells post-MI (Extended Data Fig.10c-10d). However, there was a significant reduction of VASH2 association with polymerized microtubules in *Mark4* ^-/-^ compared to wild-type cardiomyocytes (Fig.4f-4g). In conclusion, our results demonstrate that MARK4 regulates microtubule detyrosination by phosphorylating MAP4 and controlling VASH2 accessibility to the microtubules (Extended Data Fig.10e).

## Discussion

Detyrosinated microtubules impede contractile function of cardiomyocytes from failing human hearts^7^, and targeting the regulatory mechanism controlling microtubule detyrosination could represent a new inotropic strategy for improving cardiac function. We show a major role of MARK4 in the alteration of cardiomyocyte contractility through modulation of microtubule detyrosination in the ischaemic heart. It will be interesting to examine whether this protective effect of MARK4 inactivation on cardiac function after MI is sustained in the very long term (several months after MI) without inducing any harmful side effects, and whether MARK4 inhibition can improve contractile function in the setting of non-ischaemic heart failure. Furthermore, the marked improvement in relaxation kinetics in the absence of MARK4 raises the possibility of a potential beneficial effect of MARK4 inhibition in the setting of heart failure with preserved ejection fraction, an increasingly common cardiac syndrome associated with high morbidity and mortality. The molecular and structural mechanisms of MARK4 coupled with MAP4 and VASH2/SVBP in modifying microtubule detyrosination will need to be probed in other settings such as mitosis where regulation of detyrosinated microtubules has significant pathophysiological relevance^9,36^, and the differential role of other TCPs (*e.g.* VASH1) will need to be further studied in the future.

## Methods

### Mice

All *in vivo* experiments using mice were approved by the Home Office, UK, and were performed under PPL PA4BDF775. All mice were on a C57BL/6 background and housed under standard temperature (18-23°C) and humidity (40-60%), with 12-hour light/dark cycle. *Mark4* ^-/-^ mice were kindly provided by Prof Yuguan Shi^24^ (Barshop Institute), and Mutant Mouse Resource and Research Center (MMRRC, University of California, Davis); *aMHC-mcm* ^+/-^ Cre mice were originally from the Jackson Laboratory; *Mark4* ^fl/fl^ mice were from Taconic Biosciences. *aMHC-mcm* ^+/-^ Cre mice were crossed with *Mark4* ^fl/fl^ mice to generate *aMHC-mcm* ^+/-^ Cre; *Mark4* ^fl/fl^. The Cre-mediated excision of floxed *Mark4* alleles was induced by treatment with tamoxifen dissolved in corn oil for intraperitoneal injection (i.p.) at 20mg/kg (body weight) per day for 5 constitutive days.

### LAD coronary artery ligation model

Permanent left anterior descending coronary artery ligation was performed on experimental animals as described^22,23^ previously with minor modification. Mice, at 8-10 weeks of age, were anesthetized using ketamine at 100mg/kg (body weight) and xylazine at 10mg/kg (body weight) via i.p., and then intubated and ventilated with air (supplemented with oxygen) using a small-animal respirator. A thoracotomy was performed in the fourth left intercostals space. The left ventricle was visualized and the pericardial sac was ruptured to expose the LAD. The LAD was permanently ligated using a 7-0 Prolene suture. The suture was passed approximately 2mm below the tip of the left auricle. Significant colour changes at the ischaemic area and ECG changes were monitored as an indication of successful coronary artery occlusion. The thoracotomy was closed with 6-0 Prolene sutures. Sham-operated animals underwent the same procedure without coronary artery ligation. The endotracheal tube was removed once spontaneous respiration resumed, and the mice were placed on a warm recovery cage maintained at 37 °C until they were completely awake. At the indicated time points in the experimental timeline, the mice were sacrificed by CO_2_ asphyxiation, and the tissues were subsequently harvested for analysis.

### Bone marrow transplants

Eight to ten-week old C57BL/6 mice were maintained overnight with Baytril (Bayer AG) before irradiation with two doses of 5.5 Gy (separated by 4 hours) followed by reconstitution with 1×10^7^ sex-matched donor bone marrow cells. Animals were randomly assigned to receive the *Mark4* ^-/-^ or *Mark4* ^+/+^ bone marrow. Mice were then maintained on Baytril for a 4-week recovery period before performing LAD ligation.

### Echocardiography

Transthoracic echocardiography was performed on all mice using Vevo 3100 with a MX400 linear array transducer (VisualSonics), 30 MHz. Mice were anesthetized with 2-3% isoflurane and kept warm on a heated platform (37 °C). The chest hairs were removed using depilatory cream and a layer of acoustic coupling gel was applied to the thorax. After alignment in the transverse B-mode with the papillary muscles, cardiac function was measured on M-mode images. Echocardiography data were collected by using VisualSonics Vevo 3100, and analyzed by using Vevo LAB3.1.1.

### Histological analysis

Whole hearts were excised at different time point after LAD ligation, rinsed in PBS and fixed with 4% PFA overnight at 4°C. Fixed tissues were thoroughly washed in PBS, and then sinked in 30% sucrose. Tissues were embedded and sectioned by a cryostat into 10μm thick slices, which started at the apex and ended at the suture ligation site. Masson’s trichrome staining was performed to determine scar size. Scar size (in %) was calculated as total infarct circumference divided by total left ventricle circumference. Some hearts were excised at 24 hours post-MI and quickly sliced into four 1.0 mm thick sections perpendicular to the long axis of the heart. The sections were then incubated with 1% triphenyltetrazolium chloride (TTC, Sigma) for 15 minutes at 37°C and then digitally photographed. For infarct size at 24 hours post-MI, TTC-stained area, and TTC-negative staining area (infarcted myocardium) were measured using ImageJ (v2.0). Myocardial infarct size was expressed as a percentage of the total left ventricle area. Images were obtained by using Leica DM6000 B Microscope, collected by using LAS AF software (2.4.0 build 6254), and analyzed by using ImageJ (v2.0) analyze tools.

### Tissue immunohistochemistry

Whole hearts were excised, quickly washed in PBS, and flash frozen. Tissues were then embedded and cryo-sectioned. Slices were fixed in pre-chilled methanol for 10 minutes at - 20°C. After washing with PBST (0.1% tween-20 in 1 × PBS), slices were incubated with 3% H_2_O_2_ (in PBS) for 10 minutes, and then with blocking buffer (5% BSA in PBST) for 1 hour at room temperature. The primary antibody against MARK4 (Abcam, ab124267, used at 1:200), or rabbit IgG isotype control (Novus Biologicals, NB810-56910, used at 1:1000) was used for overnight at 4°C. Extensive washing steps were performed to remove nonspecific binding antibody. Slices were incubated with the biotinylated secondary antibody (Abcam, ab6720, used at 1:800) for 1 hour at room temperature. Reagents A and B from Avidin-Biotin Complex kit (VECTOR, PK-4000) were diluted and added to the slides. The slides were stained with ImmPACT DAB peroxidase substrate (VECTOR, SK-4105), and counterstained with hematoxylin. Images were obtained by using Leica DM6000 B Microscope, collected by using LAS AF software (2.4.0 build 6254), and analyzed by using ImageJ (v2.0) analyze tools.

### Microtubule co-sedimentation assay

Lyophilized porcine brain tubulin (T240) was purchased from Cytoskeleton, Inc (Denver, USA). Recombinant proteins of 4R-MAP4 and VASH2/SVBP were previously described^12,34^. The desiccated tubulin was reconstituted in the microtubule polymerization buffer to 10 mg/mL. To generate polymerized microtubules, tubulin was diluted to 2 mg/mL in the polymerization buffer (80mM K-PIPES, pH 6.8, 1mM MgCl2, 1mM EGTA and 1mM DTT), supplemented with 5% glycerol and 1mM GTP at 37°C for 30 minutes, and then stabilized by incubating with 2.5 μM taxol at 37°C for 15 minutes. The taxol stabilized MTs were centrifuged over cushion buffer (polymerization buffer with 40% glycerol) at 131,700g at 37°C for 15 minutes to remove the free tubulin. The pellet was suspended in the polymerization buffer with 1 μM taxol. Taxol influenced the association of 4R-MAP4 with the MTs in our assay. 4R-MAP4 association was facilitated when taxol was completely excluded from the buffer. The MTs without taxol were susceptible to depolymerisation if stored at room temperature. In these conditions, the polymerized microtubules were maintained at 37°C throughout the experiment. For the co-sedimentation assay, the MTs were mixed with various concentrations of 4R-MAP4 (1-6 μM) and VASH2/SVBP (1-4 μM) in the polymerization buffer. In the competition experiments, the MTs were incubated with specified 4R-MAP4 concentrations (1-4 μM) for 10 minutes, followed by addition of constant amount of VASH2/SVBP (3 μM) with further incubation of 10 minutes. Subsequently, the reaction mixture was centrifuged in TLA120.2 rotor at 55,000 rpm for 15 minutes. The pellet fraction containing the MTs and bound proteins was resuspended in the loading buffer. The samples were loaded on 10 % SDS-PAGE gel and stained with Colloidal Coomassie blue dye (ThermoFisher). The experiments were repeated at least 3 times. The band intensities were analyzed using ImageJ (v2.0).

### Murine cardiomyocyte isolation

Cardiomyocytes preparation was accomplished as previously described^28^. In brief, mice were anesthetized, and the chest was opened to expose the heart. Descending aorta was cut, and the heart was immediately flushed by injection of 7 mL EDTA buffer into the right ventricle. Ascending aorta was clamped, and the heart was transferred to a 60 mm dish containing fresh EDTA buffer. Digestion was achieved by sequential injection of 10 mL EDTA buffer (NaCl, 130mM; KCl, 5mM; NaH_2_PO_4_, 0.5mM; HEPES, 10mM; Glucose, 10mM; BDM, 10mM; Taurine, 10mM; EDTA, 5mM; pH to 7.8), 3 mL perfusion buffer (NaCl, 130mM; KCl, 5mM; NaH2PO4, 0.5mM; HEPES, 10mM; Glucose, 10mM; BDM, 10mM; Taurine, 10mM; MgCl2, 1mM; pH to 7.8), and 30 to 50 mL collagenase buffer (Collagenase 2, 0.5mg/mL; Collagenase 4, 0.5mg/mL; Protease XIV, 0.05mg/mL; made fresh and diluted in perfusion buffer) into the left ventricle. Left ventricle was then separated and gently pulled into 1 mm pieces using forceps. Cellular dissociation was completed by gentle trituration, and enzyme activity was inhibited by addition of 5 mL stop buffer (Perfusion buffer containing 5% sterile FBS). Cell suspension was passed through a 100-μm filter. Cells underwent 4 sequential rounds of gravity settling, using 3 intermediate calcium reintroduction buffers (Buffer 1: 75% Perfusion buffer with 25% culture media; Buffer 2: 50% Perfusion buffer with 50% culture media; Buffer 3: 25% Perfusion buffer with 75% culture media; Culture media comprise 0.1% BSA, 1% ITS, 10mM BDM, 1% CD lipid and 5% Penicillin / Streptomycin in M199) to gradually restore calcium concentration to physiological levels.

### Primary cardiomyocyte culture and adenoviral transduction

Adenoviral vectors including **pAdeno-SV40-GFP-Blank** vector (Adv-null), **pAdeno-Ttl-SV40-GFP** vector (Adv-Ttl) (NM_027192.2) and **pAdeno-Mark4-SV40-GFP** vector (Adv-Mark4) (NM_172279.1) were purchased from Applied Biological Materials Inc. Adenoviral vector **pAV[shRNA]-EGFP-U6>mMap4** (**s**hRNA *Map4* target sequence: AGAGTGGACTATCCGGATTAT), adenoviral vector **pAV[shRNA]-EGFP-U6>mVash2** (**s**hRNA *Vash2* target sequence: GAGAATCCTTGCCTATCAAAT), and adenoviral vector **pAV[shRNA]-EGFP-U6>Scramble** were purchased from VectorBuilder. 6 well plates or coverslips were coated with laminin at a final concentration of 5 μg/mL in PBS overnight at 4°C. The wells were washed and air-dried for 10 minutes before plating cells. After collecting the cells by gravity settling and calcium re-introduction, the final myocyte pellets were re-suspended in 2 mL culture media and 2 mL pre-equilibrated plating media (0.1% FBS, 10mM BDM, and 5% Penicillin / Streptomycin in M199) for culture. After one-hour incubation, cell media was changed with pre-equilibrated culture media and adenovirus vectors were administered at 5*10^6^ pfu/mL. After co-culture with virus for 8 hours, fresh culture media was used to wash and replace the old culture media with virus. Cells were either subjected to contractility assay and western blotting immediately after media change (in the experiments of overexpression of TTL), or collected at 48 hours after transduction (in the experiments of overexpression of *Mark4*, shRNA *Map4* and shRNA *Vash2*) for the subsequent assays.

### Cardiomyocyte contractility assay

Sarcomere shortening and relaxation were measured in freshly isolated left ventricular cardiomyocytes of murine hearts using the integrated IonOptix contractility/photometry system. Cardiomyocytes were maintained in normal Tyrode’s solution (NaCl, 140mM; MgCl_2_, 0.5 mM; NaH_2_PO_4_, 0.33mM; HEPES, 5mM; glucose, 5.5mM; CaCl_2_, 1mM; KCl, 5mM; NaOH, pH to 7.4) at room temperature, electrically stimulated at 2 Hz using a field stimulator, and changes in sarcomere length were recorded. Basal and peak sarcomere length, maximum departure/return velocities and time to peak were measured. All measurements were performed at room temperature. For PTL experiments, cardiomyocytes were treated with 10 μM PTL (Sigma P0667) or vehicle at room temperature in normal Tyrode’s solution for 1 hour before contractility measurements, and the vehicle dimethyl sulfoxide (DMSO) diluted in the same way was applied as control. All measurements were performed at room temperature within 4 hours. Data were collected and analyzed by using IonWizard 7.4.

### Calcium measurement

Measurement of intracellular calcium was performed in freshly isolated left ventricular cardiomyocytes using integrated IonOptix contractility/photometry system. Cardiomyocytes were loaded with 1 μM Fura-2-AM for 20 minutes (protected from light), and then washed to allow de-esterification for 20 minutes. Cells were then rinsed with a normal Tyrode’s Solution. Cells were stimulated at 2 Hz using a field stimulator with dual excitation (at 360 and 380 nm), and emission light was collected at 510 nm. Changes in calcium transients were recorded using IonOptix software. All the cells analyzed were beating. All measurements were performed at room temperature within 4 hours. Data were collected and analyzed by using IonWizard 7.4

### Immunofluorescence and image acquirement

Cardiomyocytes were fixed with pre-chilled methanol for 10 minutes, then washed twice using PBST (0.1% tween-20 in 1×PBS) with 5 minutes intervals. Cells were blocked for 1 hour at room temperature with blocking buffer (5% BSA in PBST) and incubated with primary antibodies overnight at 4°C. The primary antibodies were: Detyrosinated α-tubulin (Abcam, ab48389, used at 1:200), α-tubulin (CST, 3873S, used at 1:200), MARK4 (Abcam, ab124267, used at 1:200), APC anti-mouse CD45 (BioLegend, 103112, used at 1:200) and rabbit IgG isotype control (Novus Biologicals, NB810-56910, used at 1:2000). The cells were then washed with PBST and incubated with secondary antibody for 1 hour at room temperature. The secondary antibodies were: AF488 donkey anti-rabbit IgG (Invitrogen, A21206, used at 1:200), AF647 goat anti-mouse IgG (Invitrogen, A21236, used at 1:200), AF647 goat anti-rat IgG (Invitrogen, A21247, used at 1:200). DAPI (Sigma, 10236276001, used at 1:1000) was used. Confocal images were obtained by Leica SP5 Confocal Laser Scanning Microscope, collected by LAS AF software (2.7.3.9723), and analyzed by ImageJ (v2.0) analyze tools.

### STED imaging and image analysis

Cardiomyocytes on coverslips were fixed with 100% methanol for 15 minutes at room temperature and then washed three times with PBS (5 minutes intervals). Cells were blocked with buffer (5% BSA and 0.2% TX-100 in PBS) for 30 minutes, then incubated with primary antibodies (diluted in blocking buffer) overnight at 4°C. The primary antibodies were VASH2 (Abcam, ab224723, used at 1:200), MAP4 (Abcam, ab245578, used at 1:200) and α-tubulin (CST, 3873S, used at 1:200). The cells were washed three times using wash buffer (0.05% TX-100 in PBS) at room temperature, then incubated with the secondary antibody for 1 hour at room temperature. The secondary antibodies were: Atto 594 goat anti-Rabbit IgG (Sigma, 77671, used at 1:500), and Atto 647N goat anti-mouse IgG (Sigma, 50185, used at 1:500). Cells were then washed three times in wash buffer. Cells were fixed (3% Paraformaldehyde and 0.1% glutaraldehyde diluted in PBS) followed by three washes in PBS. The coverslips were then mounted on the slide.

STED imaging was carried out on a custom multicolour system with three pulsed excitation lines, one fixed depletion line, fast 16 kHz beam scanning and gated detection centered around an Olympus IX83 microscope base. This system uses identical hardware, and a closely matched optical arrangement, to the system previously published by Bottanelli and co-workers^35^. In brief, two-colour STED imaging was performed sequentially. Images were acquired with a 100X oil immersion objective lens (Olympus, UPLSAPO 100XO/PSF). Fields of view between 23 and 27 μm^2^ were imaged with a 1024 x 1024 image format and an approximately 20 nm pixel size. Excitation powers were between 15 and 30 μW at the microscope side port while STED depletion power was approximately 120 mW at the microscope side port. Fast, 16 kHz, unidirectional beam scanning with blanking was employed to minimize light exposure. Each line of an image was scanned 850 times resulting in an image acquisition time of approximately 54 seconds per colour. STED image data were collected with a custom program written in National Instrument (NI) LabVIEW 2014 64-bit, NI FPGA Module and NI Vision Development Module.

MAP4 oligomerized puncta (with diameter longer than 400 nm) were measured and calculated using ImageJ (v2.0). The number of puncta was normalized against the cell area on each image.

For the acquired images, a dynamic thresholding algorithm was used for the image analysis. Images were converted into HSV colour images (C) with information of Hue (h), Saturation (s), and Value (v). C (I, j) was assumed as a non-background image pixel, N was the total number of non-background image pixels. The average of all the non-background image pixels was calculated as: $k=(\Sigma_{i=1}^{h}\Sigma_{j=1}^{w}C(i,j))/N$. The following three thresholds were applied to discriminate signals: h = [0,180]; s = [0,43]; v = [k+30,220]. The Gaussian filter $\left(f(x)=\frac{1}{2\pi \sigma^{2}}e^{-\frac{x^{2}+y^{2}}{2\sigma^{2}}}\right)$, a 2-D convolution operator, was used to remove noise. For the VASH2 signals, the Gaussian filter with the kernel of 3*3 was used for image denoising. For the linear microtubule signals, the Gaussian filter with the kernel of 5*5 was used for image denoising when k >= 35, and kernel of 3*3 was applied when k<35. The total numbers of VASH2 (v) and TUBULIN (t) pixels were calculated. The total number of the overlapping image pixels (o) between VASH2 and TUBULIN was calculated as VASH2 signals on the microtubules, and (1-o)/v was calculated as VASH2 signals off the microtubules. The Pearson correlation coefficient (PCC) $\left(\rho_{X,Y}=\frac{\mathrm{cov}(X,Y)}{\sigma_{X}\sigma_{Y}}\right)$ between VASH2 signals and TUBULIN signals was calculated. Automatic image processing was coded using a custom algorithm in python 3.7.8.

### Subcellular fractionations of the primary cardiomyocytes

Subcellular fractionations on primary cardiomyocytes were performed according to manufacturer’s instructions (Pierce, 87790). Briefly, cells were incubated with Cytoplasmic Extraction Buffer (CEB) which selectively permeabilizes the cell membrane for 10 minutes at 4°C with gentle mixing. Cells were centrifuged for 5 minutes at 500g and supernatants were collected. The cytoskeletal binding proteins were isolated in the Pellet Extraction Buffer (PEB).

Subcellular fractionations of primary cardiomyocytes were also obtained using a conventional method as the following: Primary murine cardiomyocytes were isolated and homogenized in pre-warmed (37°C) microtubule stabilizing buffer (MTSB buffer: PIPES, 80mM; MgCl2, 1mM; EGTA, 1mM; 0.5% Triton X-100; 10% glycerol; GTP, 0.5mM; Halt^Tm^ Protease Inhibitor Cocktail from Thermo Fisher Scientific 1862209; pH to 6.8) using Dounce homogenizer. The homogenates were centrifuged at 100,000g for 15 minutes at room temperature. The supernatants were collected as F1 (free tubulin fraction), and the pellets were dissolved in the microtubule destabilizing buffer (MTDB buffer: Tris-HCl, 20mM; NaCl, 150mM; 1% Triton X-100; CaCl_2_, 10mM; Halt^Tm^ Protease Inhibitor Cocktail from Thermo Fisher Scientific 1862209; pH to 7.4) for further incubation on ice for one hour to depolymerize the microtubules. The dissolved lysates were centrifuged at 12,000g for 15 minutes at 4°C. The pellets were incubated with 150 units micrococcal nuclease (100 units/μL, Thermo Fisher Scientific, 88216) in the MTDB buffer for 15 minutes at room temperature, and then centrifuged at 12,000g for 5 minutes at 4°C to remove the nuclear. The collected pellets were dissolved in 2xSDS buffer (4% SDS; 20% glycerol; Tris-HCl, 0.25M; pH to 6.5). The dissolved lysates were then centrifuged at 14,000g for 5 minutes at 4°C. The supernatants were collected as F2 (extraction from the stable pellet fraction), and the residual pellets were kept.

### Western blotting

The heart tissues were grounded thoroughly with a mortar and pestled in liquid nitrogen. Tissue powder was lysed using Triton lysis buffer [20mM Tris-HCl, pH to 7.5; 150mM NaCl; 1mM Na2EDTA; 1mM EGTA; 1% Triton; 1mM Na3VO4; 5mM NaF; protease inhibitor cocktail (ThermoFisher, 1862209)]. The supernatant (soluble fraction) was collected, and the pellets (insoluble fraction) were dissolved in 8M Urea (Fig.1b; Fig.3a; Fig.3d; n=12 mice in *Mark4* ^+/+^ MI group, and n=9 mice in *Mark4* ^-/-^ Fig.3b-3c). For some experiments (n=8 mice per group used for Fig.3b-3c), heart tissues were homogenized in the lysis buffer [0.1M PIPES pH to 6.8; 2mM EGTA; 0.1mM EDTA; 0.5 mM MgCl2; 20% glycerol; 0.1% Triton X-100; protease inhibitor cocktail (ThermoFisher, 1862209)], and incubated for 30 minutes at 37°C. After centrifugation (21,100g for 5 minutes), the supernantants were collected as soluble fraction, and the pellets were dissolved in the buffer [RIPA buffer (CST, 9806); 0.8% SDS; and protease inhibitor cocktail (ThermoFisher, 1862209)] and collected as insoluble fraction. Protein concentration was determined by BCA^TM^ protein assay kit (ThermoFisher, 23235). Molecular weight markers (ThermoFisher, LC5603, LC5925) were used. Supernatant samples were prepared in NuPAGE LDS sample buffer (Invitrogen) and run on NuPAGE 4-12% Bis-Tris gels (Invitrogen). Pellet samples were prepared in Tris-Glycine SDS sample buffer (Invitrogen) and run on Novex 4-20% Tris-Glycine gels (Invitrogen). All samples were blotted onto a PVDF membrane after electrophoresis. The following primary antibodies were used in the experiments: MARK4 (CST, 4834S, used at 1:1000), GAPDH (CST, 5174S, used at 1:1000), DESMIN (R&D, AF3844, used at 1:1000), detyrosinated α-tubulin (Abcam, ab48389, used at 1:200), polyglutamylated α-Tubulin (AdipoGen, AG-20B-0020-C100, used at 1:1000), acetylated α-Tubulin (Santa Cruz Biotechnology, sc23950, used at 1:1000), α-tubulin (CST, 3873S, used at 1:200). After antibody detections, membranes were revealed with ECL. Quantification of western blot band was analyzed by ImageJ (v2.0).

For the fractionation assay, equal amounts of total protein (20 μg) from each fraction were used for western blot. *DC* ^TM^ protein assay kit (Bio-Rad, 5000111) was used to measure protein concentration. Across different gels, equal amount of molecular weight marker (ThermoFisher, LC5603) was loaded in each gel. Samples were run on NuPAGE 4-12% Bis-Tris gels (Invitrogen) and blotted onto a PVDF membrane.

Some samples of CEB fraction from fractionation assay were prepared for native gel running as the following. Samples were processed in Tris-Glycine native sample buffer (ThermoFisher, LC2673) before loading without heating and adding any reducing reagent. Samples were loaded in 3-8% NuPAGE Tris-Acetate gel (ThermoFisher, EA0375BOX) for electrophoresis in Tris-Glycine native running buffer (Tris Base, 25mM; Glycine, 192mM; pH to 8.3). Native molecular marker (ThermoFisher, LC0725) was used. After electrophoresis, proteins were transferred to PVDF membrane by transfer buffer (Bicine, 25mM; Bis-Tris, 25mM; EDTA, 1mM; pH to 7.2).

Some samples from fractionation assay were prepared with denaturing treatment by adding urea. Urea (0 M, 2 M, 4 M or 8 M) was added to the samples as indicated. Micro BCA™ protein assay kit (ThermoFisher, 23235) was used to measure protein concentrations if the samples were added with Urea. Samples were then processed in Tris-Glycine SDS sample buffer (ThermoFisher, LC2676) and reducing reagent (10% 2-mercaptoethanol). 4-20% Tris-Glycine gel (ThermoFisher, EC6026BOX) was used for electrophoresis in Tris-Glycine SDS running buffer (Tris Base, 25 mM; glycine, 192 mM; 0.1% SDS; pH to 8.3). After electrophoresis, proteins were transferred to PVDF membrane by transfer buffer (Tris Base, 12 mM; glycine, 96 mM; pH to 8.3).

The primary antibodies used for fractionation assays were: Detyrosinated α-tubulin (Abcam, ab48389, used at 1:1000), α-tubulin (CST, 3873S, used at 1:1000), TTL (Proteintech, 13618-1-AP, used at 1:1000), VASH1 (Abcam, ab199732, used at 1:1000), VASH2 (Abcam, ab224723, used at 1:1000), MAP4 (phospho S1073) (Abnova, PAB15916, used at 1:1000), MAP4 (Abcam, ab245578, used at 1:1000), MAP4 (phospho S941) (Abcam, ab56087, used at 1:1000), GAPDH (CST, 5174S,used at 1:1000), DESMIN (R&D, AF3844, used at 1:1000). Membranes were revealed with ECL. Quantification of western blot band was performed using ImageJ (v2.0). The band density was normalized in two steps: 1). The density of the targeted band was first normalized against the density of the loading molecular weight marker band (Norm 1). 2). The value of Norm 1 was internally normalized against the average value of Norm1 of the control group (Norm2). The finalized value (Norm 2) was used to compare the fold changes against the value of control groups across different gels. DESMIN was used as marker for the pellet fraction, and GAPDH was used as a marker for the cytosolic fraction. Coomassie blue stained gels loaded with the same amounts of proteins as used in western blotting experiments, or Ponceau S stained membranes after the transferring step were used to confirm the equal loading. All the immunoblots, gels and membranes associated with the data presented in the Figures and Extended Data Figures are provided (Supplementary Figure 1).

### Heart tissue digestion and flow cytometry

Hearts were collected and the left ventricle was isolated, minced with fine scissors, and subjected to enzymatic digestion solution [RPMI 1640, collagenase D (0.2 mg/mL, Roche), dispase (1 U/mL, StemcellTM Technologies) and DNase I (0.2 mg/mL, Sigma)] for 45 minutes at 37°C. Cells were collected, filtered through 40-μm nylon mesh, and washed with PBS with 2.5% vol/vol fetal bovine serum. Cell suspensions were incubated with Zombie Aqua™ Fixable Viability Kit (Biolegend, 423102, used at 1:1000) for 20 minutes at room temperature then washed with PBS. Cells were then stained with fluorescently labelled anti-mouse antibodies comprised of APC anti-mouse CD45 (Biolegend, 103112, used at 1:100), AF488 anti-mouse CD11b (Biolegend, 101217, used at 1:100), Pacific blue anti-mouse Ly6G (Biolegend, 127612, used at 1:100), PE anti-mouse F4/80 (Biolegend, 123110, used at 1:100), PECY7 anti-mouse CD11c (Biolegend, 117318, used at 1:100), Brilliant Violet 605 anti-mouse CD3 (Biolegend, 100237, used at 1:100) and FITC anti-mouse CD19 (Biolegend, 553785, used at 1:100), diluted in staining buffer for 30 minutes at 4°C in the presence of 24G2 Fc receptor blocker (obtained from Division of Immunology, Department of Pathology, University of Cambridge), prior to extensive washing. The cytometric acquisition was performed on a LSR II Fortessa (BD biosciences). Cell analysis was done using BD FACSDiva Software 6.0 and FlowJo software (v10).

### Real-time PCR

For gene expression analysis, RNA from heart tissues or separated cardiomoycytes was isolated using an RNAeasy mini kit (Qiagen). Reverse transcription was performed using a QuantiTect reverse transcription kit (Qiagen). qRT-PCR was performed with SYBR Green qPCR mix (Eurogentec) using the Roche LightCycler 480II. Primer sequences are as follows: Mark4 (For. 5’-GGACACGCATGGCACATTG-3’; Rev. 5’-GCAGGAAGCGATAGAGTTCCG-3’); Vash2 (For. 5’-GCCTTCCTGGCTAAGCCTTC-3’; Rev. 5’-CCCTGTGTGGTTGTATTGTAGAG-3’); Hprt (For. 5’-TCAGTCAACGGGGGACATAAA-3’; Rev. 5’-GGGGCTGTACTGCTTAACCAG-3’); Rpl4 (For. 5’-CCGTCCCCTCATATCGGTGTA-3’; Rev. 5’-GCATAGGGCTGTCTGTTGTTTTT-3’); Rpl13a (For. 5’-AGCCTACCAGAAAGTTTGCTTAC-3’; Rev. 5’-GCTTCTTCTTCCGATAGTGCATC-3’). The average of three housekeeping genes (Hprt, Rpl4, and Rpl13a) was used as reference for qPCR gene expression analysis.

### Measurement of cTnI and inflammatory cytokines

Serum was collected within 24 hours post-MI or at day 3 post-MI. Measurements of cardiac injury biomarker (collected within 24h) and cytokines (collected at day 3 Post-MI) were performed by core biochemical assay laboratory of Cambridge University Hospitals.

### Statistics and Reproducibility

All values in the text and figures are presented as mean ± s.e.m. of independent experiments with given n sizes. Statistical analysis was performed with Prism 7.05 (GraphPad) and Excel (Microsoft Excel 2102). Violin plots were created with Prism 9.1.0 (216) (GraphPad). Data were tested for normality using a Kolmogorov-Smirnov test. Group comparisions were analyzed using two-tailed analyses. Comparisons of 3 groups or more were analyzed using one-way (one variable) or two-way ANOVA (two variables) followed by the Bonferroni post-hoc correction for multiple comparisons when appropriate. P<0.05 was considered statistically significant.

Fig.1b, Fig.1c, Fig.3a, Extended Data Fig.2a, Extended Data Fig.5a, Extended Data Fig.7b, and Extended Data Fig.8a-8b are representative figures for 3 independent experiments. Extended Data Fig.8e-8g are representative figures for 2 independent experiments.

## Extended Data

## Supplementary Material

EMS140663_Sd_ed_fig-8

EMS140663_Sd_ed_fig-10

EMS140663_Sd_ed_fig-7

EMS140663_Sd_ed_fig-5

EMS140663_Sd_ed_fig-4

EMS140663_Sd_ed_fig-2

EMS140663_Sd_ed_fig-9

EMS140663_Sd_ed_fig-3

EMS140663_SD_Fig_1

EMS140663_SD_Fig_4

EMS140663_SD_Fig_3

EMS140663_SD_Fig_2

EMS140663_Sup_Fig_1

EMS140663_Sup_info

## Data Availability

All the associated raw data presented in this paper are available from the corresponding author upon request. Source data are provided with this paper.

## Code availability

Custom codes used in this paper are available on Github (http://github.com).
