# Supplementary material for: MARK4 controls ischaemic heart failure through microtubule detyrosination
Source: Nature. Author manuscript; Available in PMC 2021 Dec 31. (PMC7612144; doi:10.1038/s41586-021-03573-5)
Supplement: EMS140663_Sup_Fig_1 [file EMS140663-supplement-EMS140663_Sup_Fig_1.pdf]

Supplementary Figure 1. Immunoblots, gels, and membranes associated with the data presented in Figures and Extended Data Figures (page 1-15).

Figure 1b

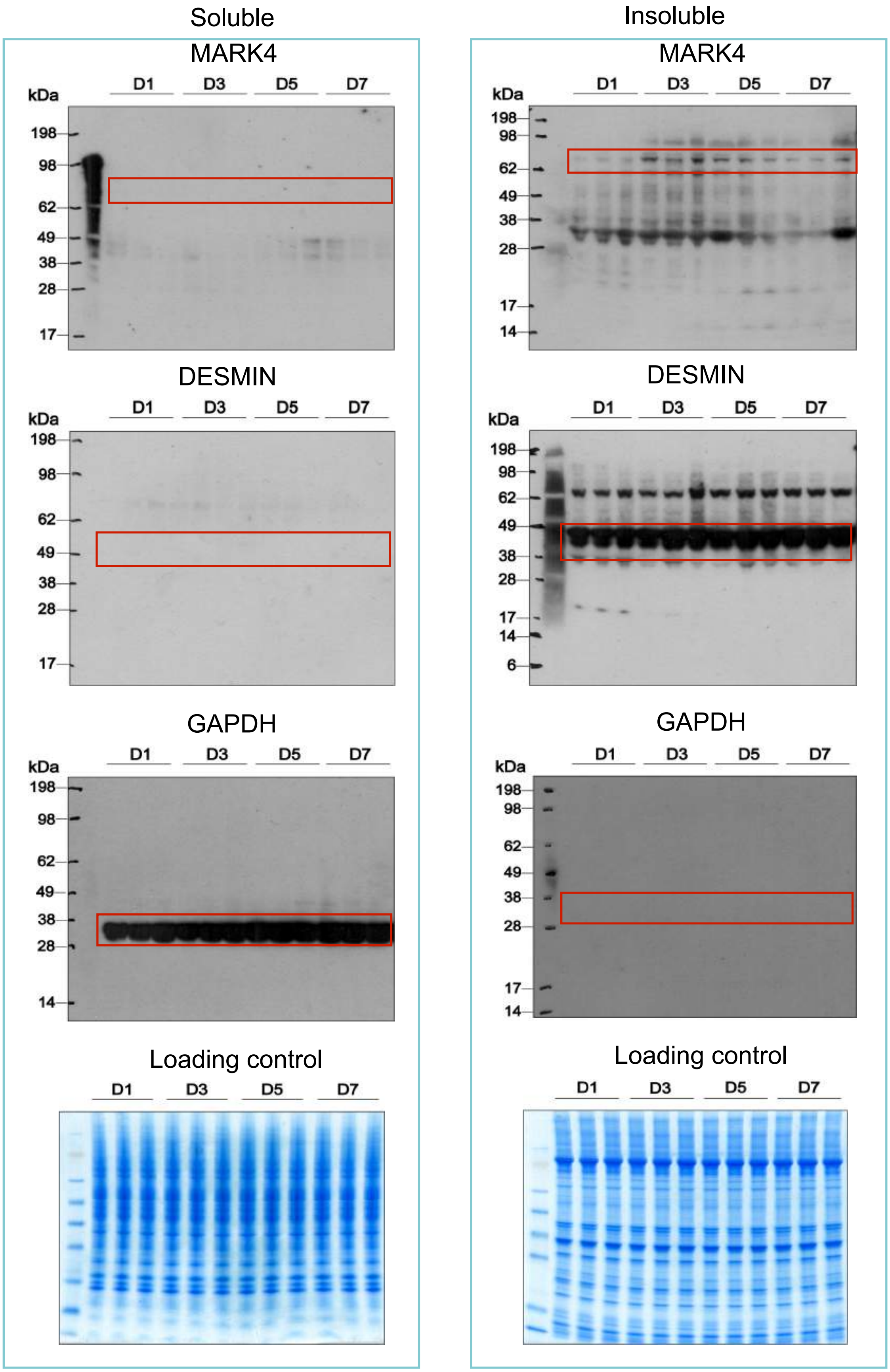
Soluble
Insoluble
MARK4
DESMIN
GAPDH
Loading control
D1
D3
D5
D7
kDa
198
98
62
49
38
28
17
14
6

Figure 3a

Soluble

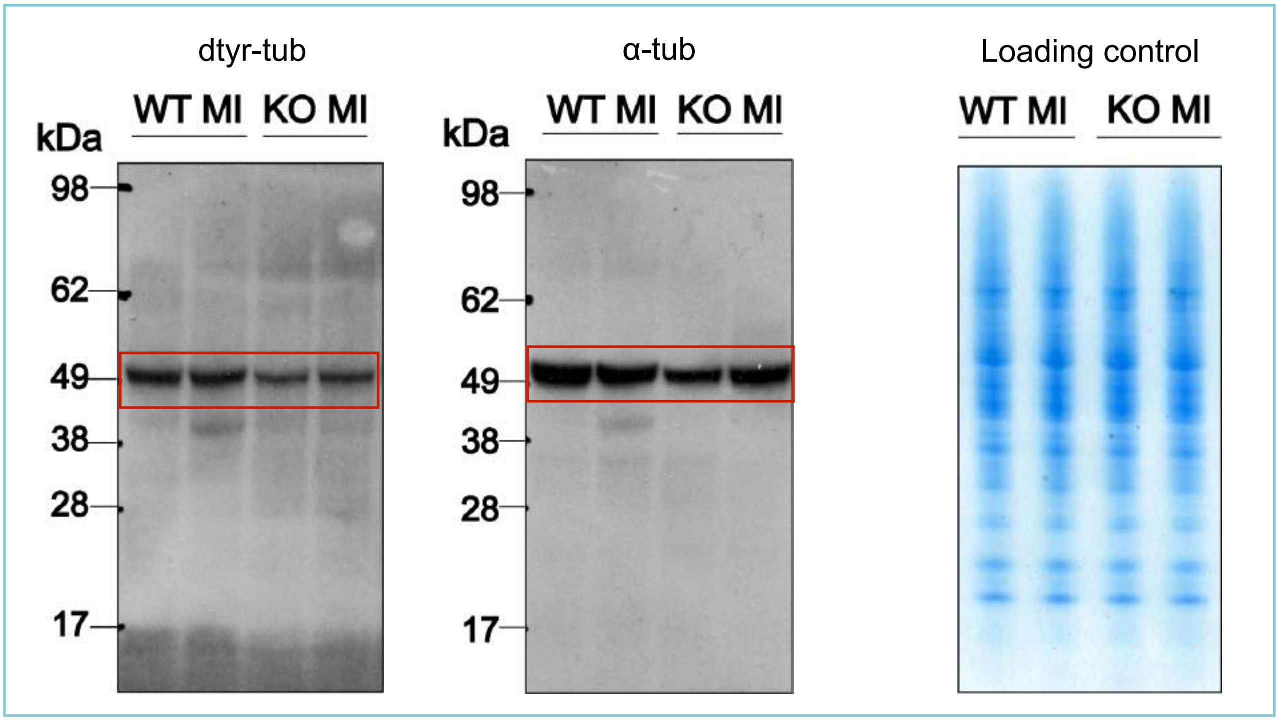

Insoluble

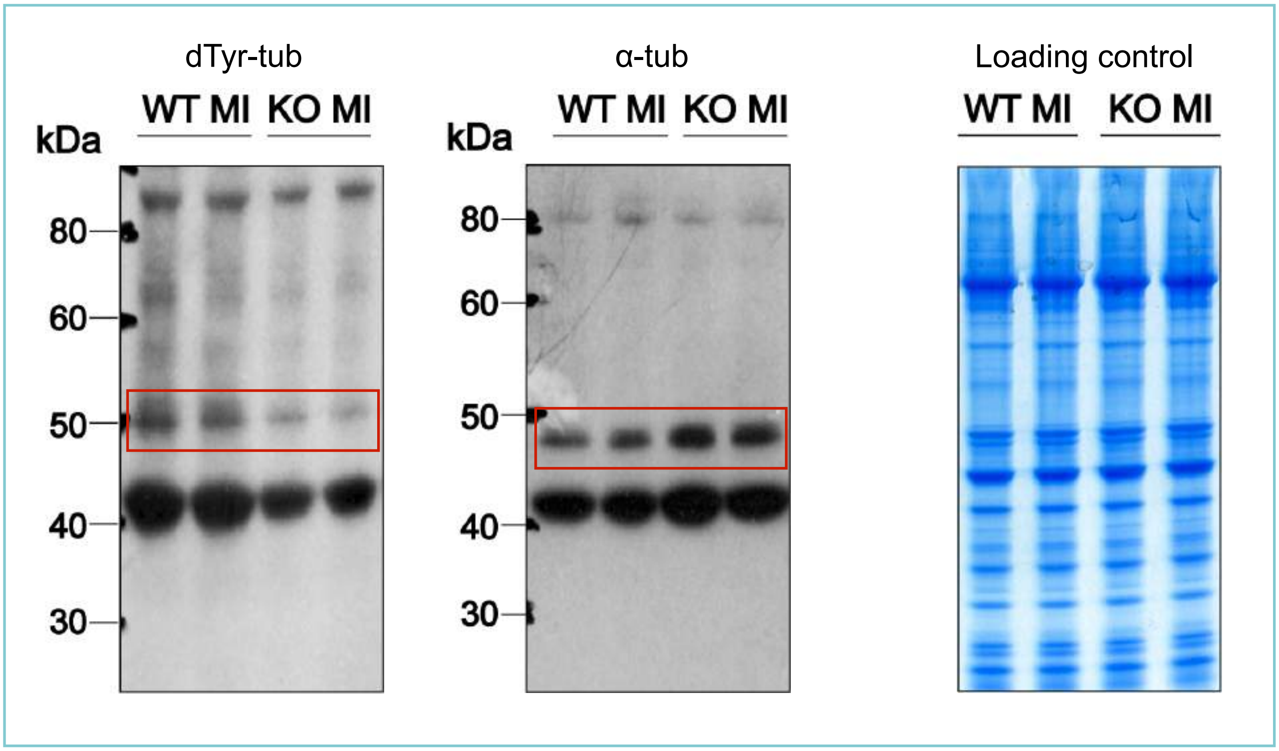

Figure 4a

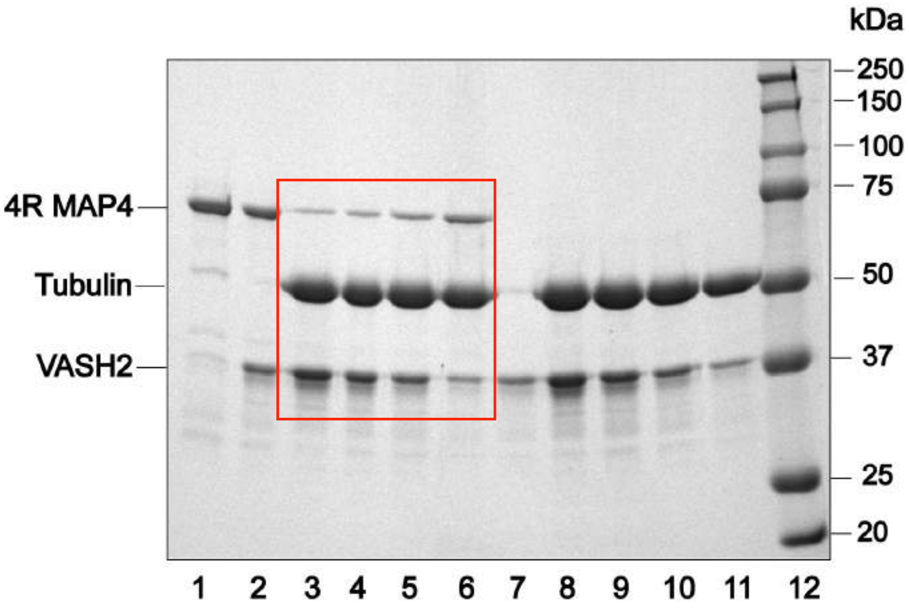

kDa
250
150
100
75
50
37
25
20
4R MAP4
Tubulin
VASH2
1 2 3 4 5 6 7 8 9 10 11 12

Figure 4c

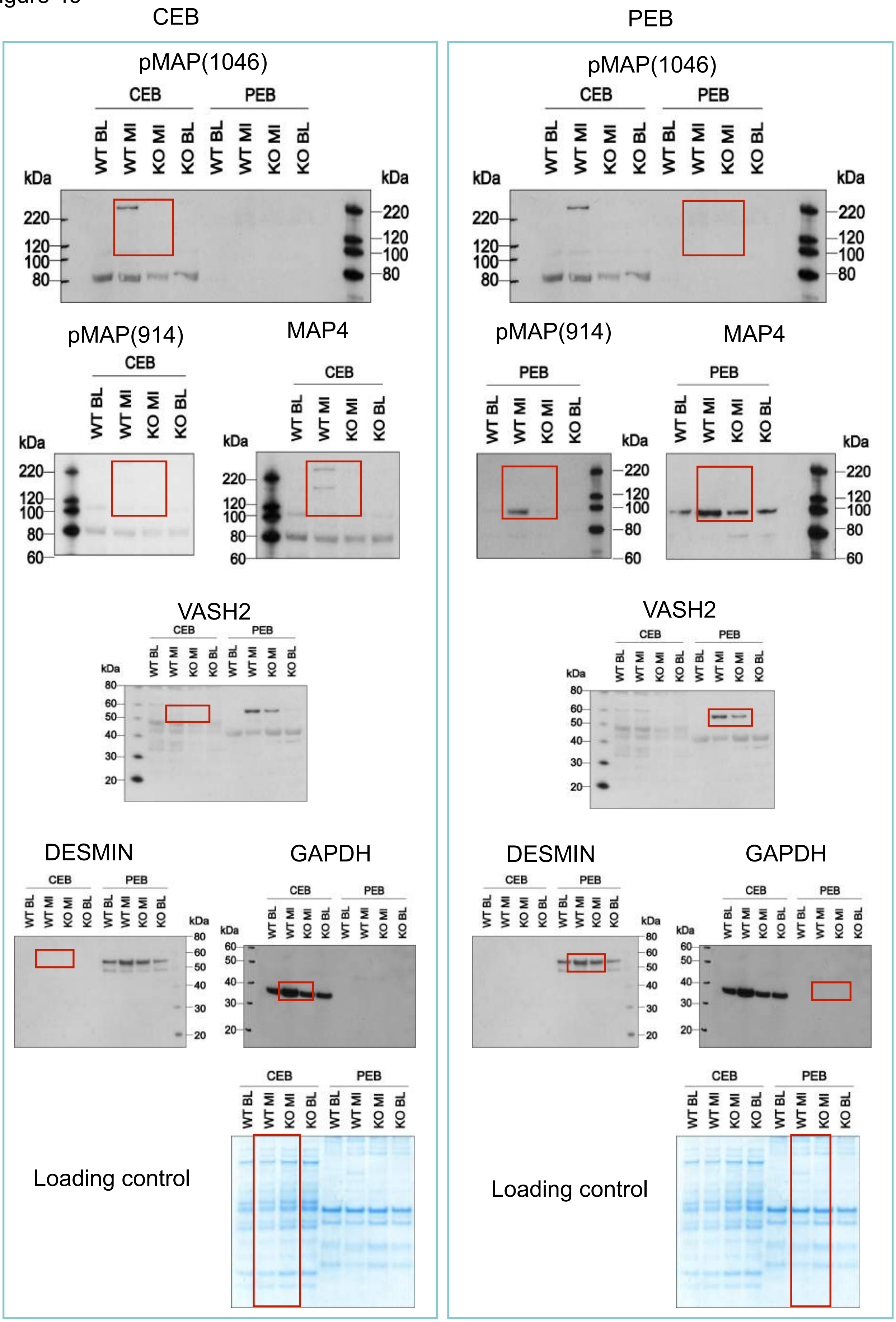
CEB
PEB
pMAP(1046)
pMAP(914)
MAP4
VASH2
DESMIN
GAPDH
Loading control
CEB
PEB
WT BL
WT MI
KO MI
KO BL
kDa

Extended Data Fig. 2d

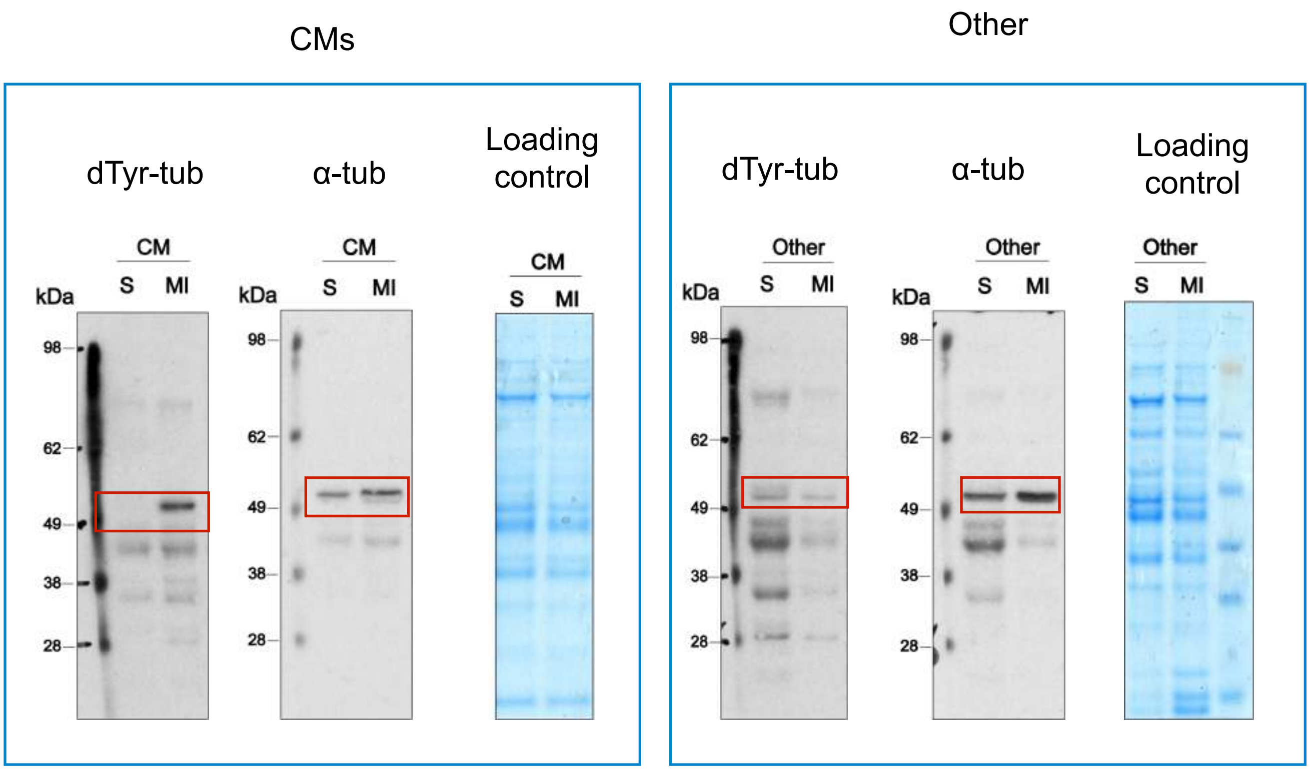

Extended Data Fig. 2f

dTyr-tub

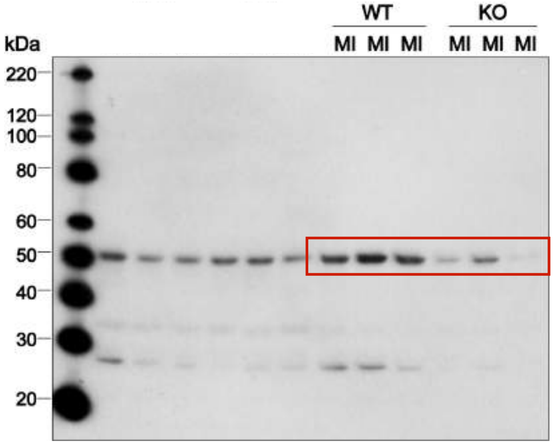

Polyglu-tub

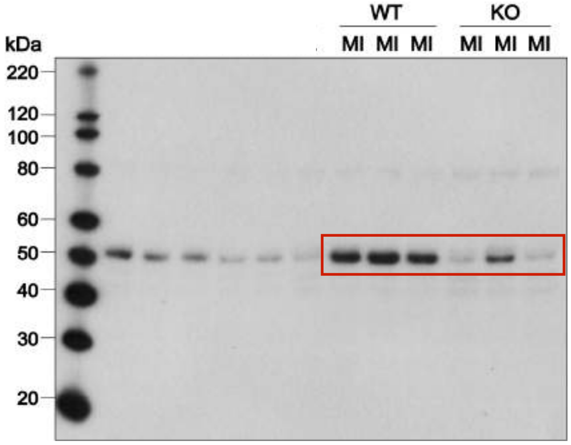

Ace-tub

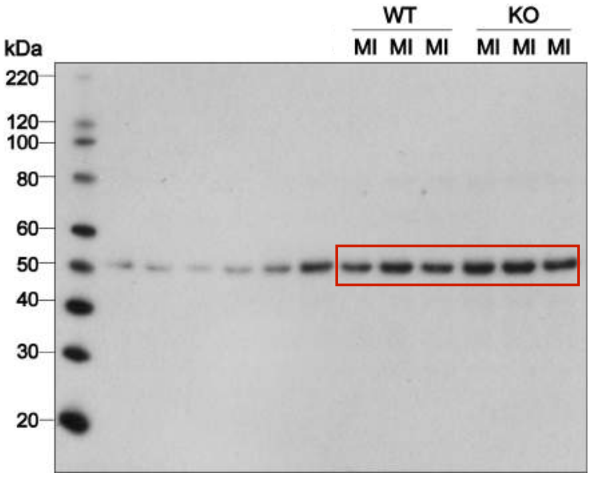

α-tub

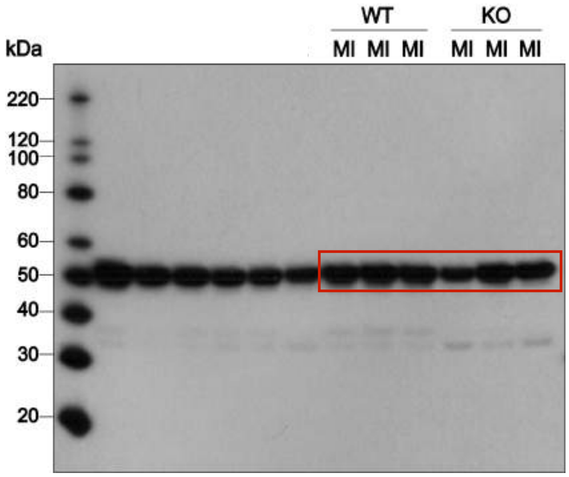

Loading control

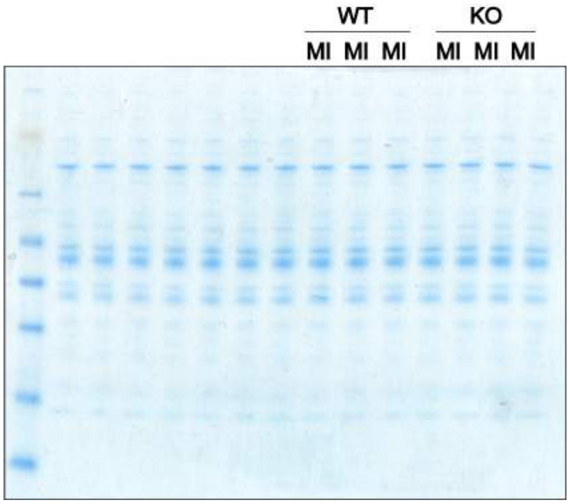

Extended Data Fig. 5a

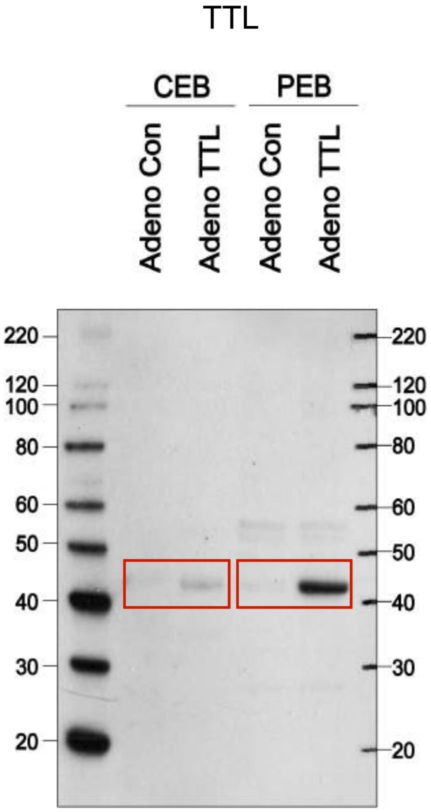

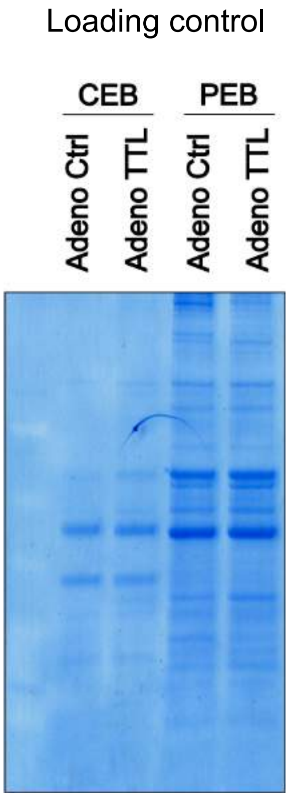

Extended Data Fig. 6c

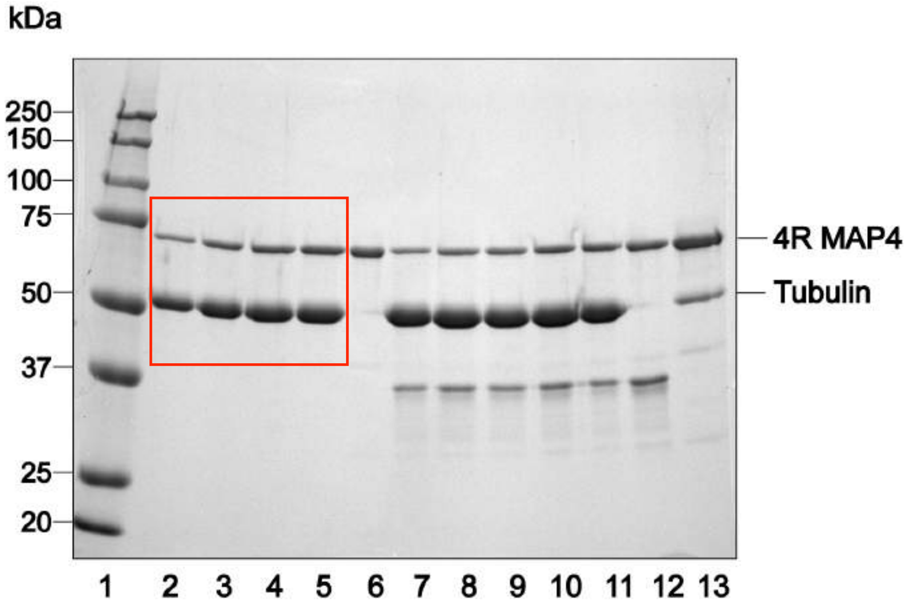

Extended Data Fig. 6e

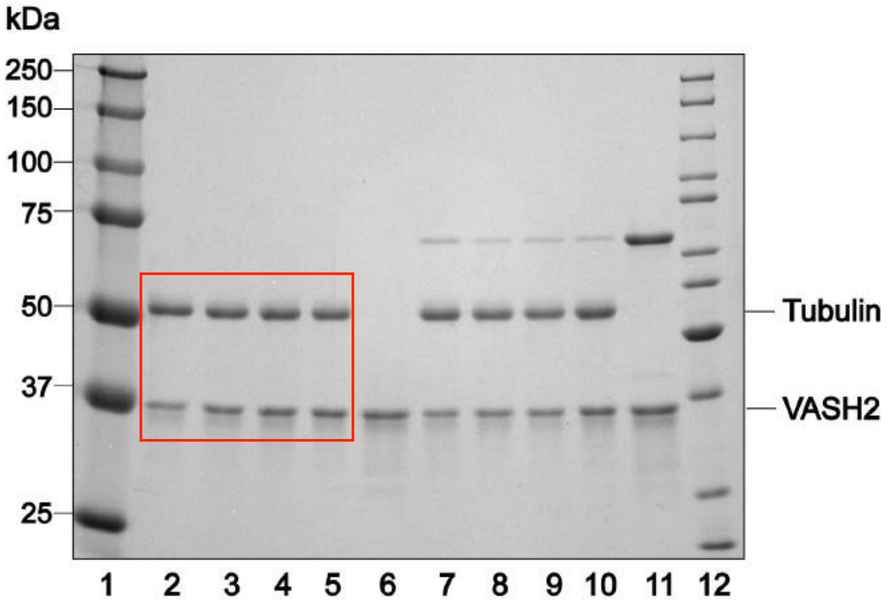

Extended Data Fig. 7b

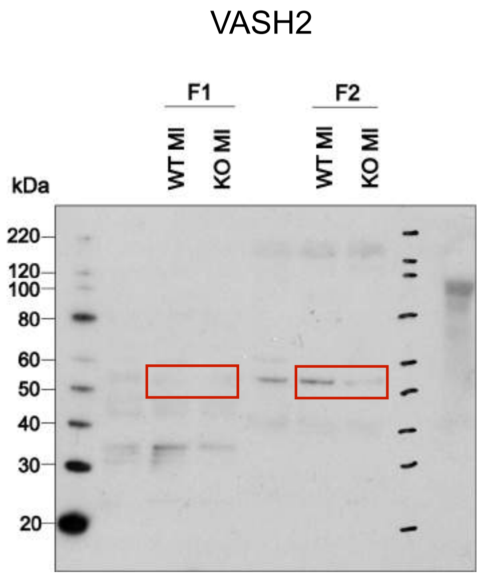

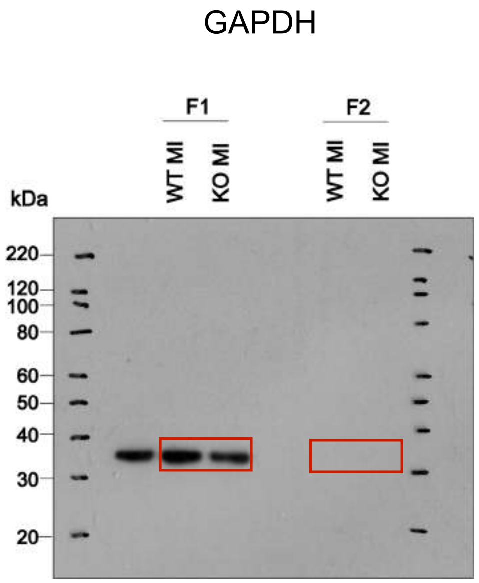

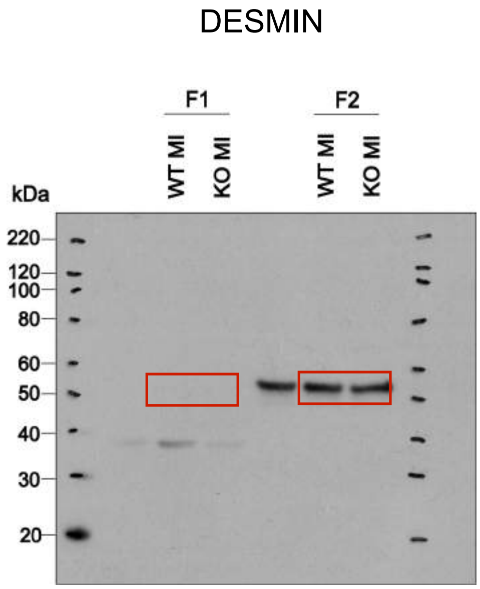

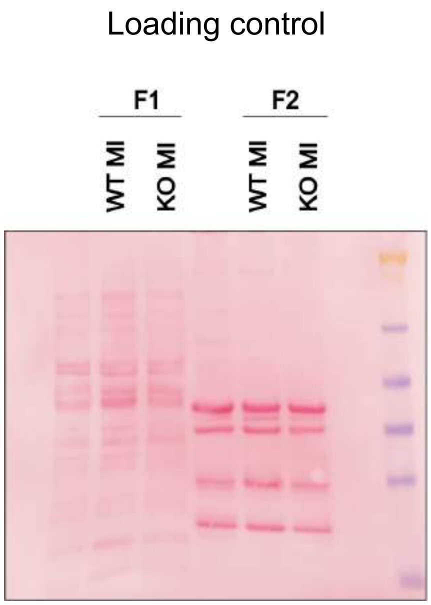

Extended Data Fig. 7c

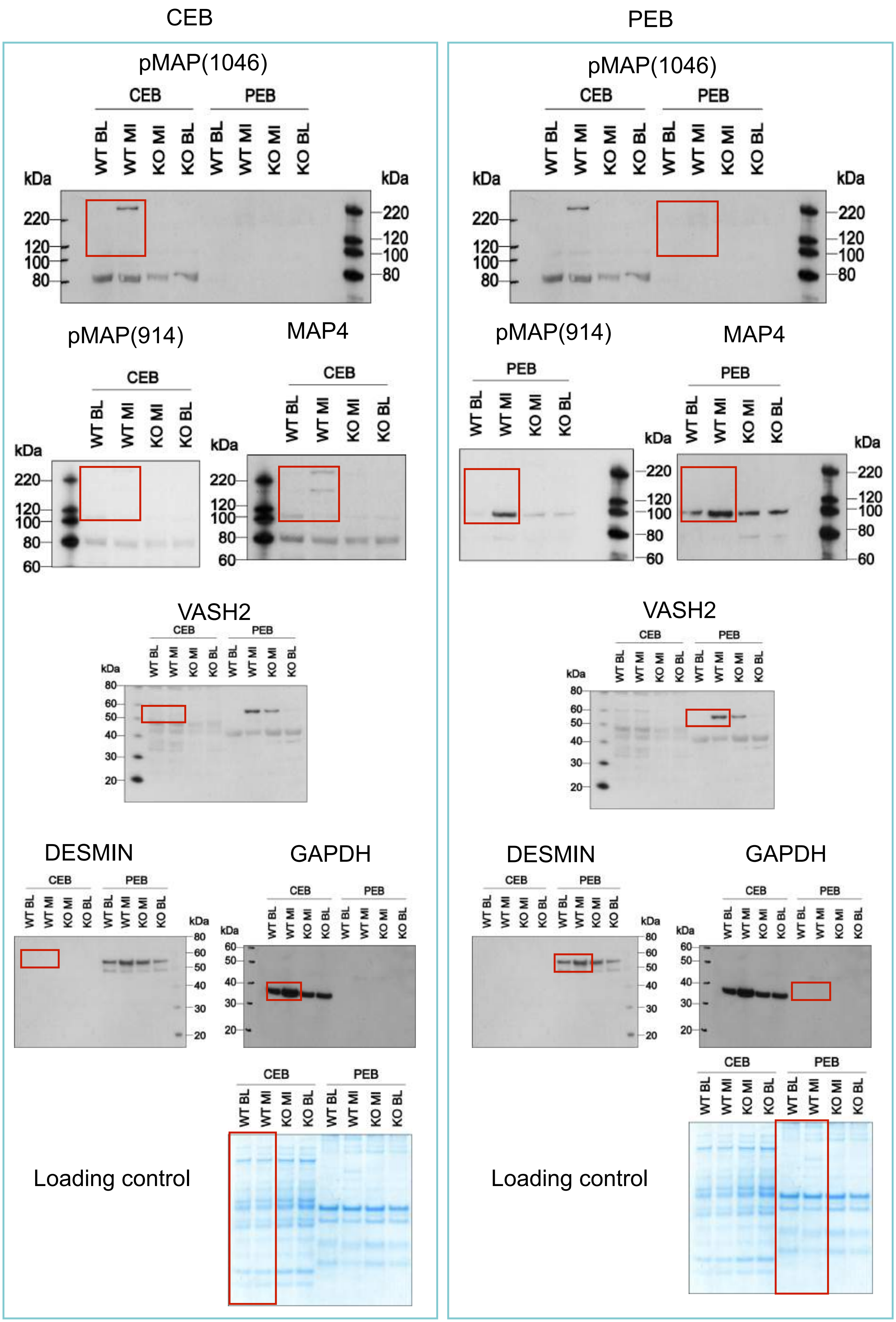

Extended Data Fig. 7f

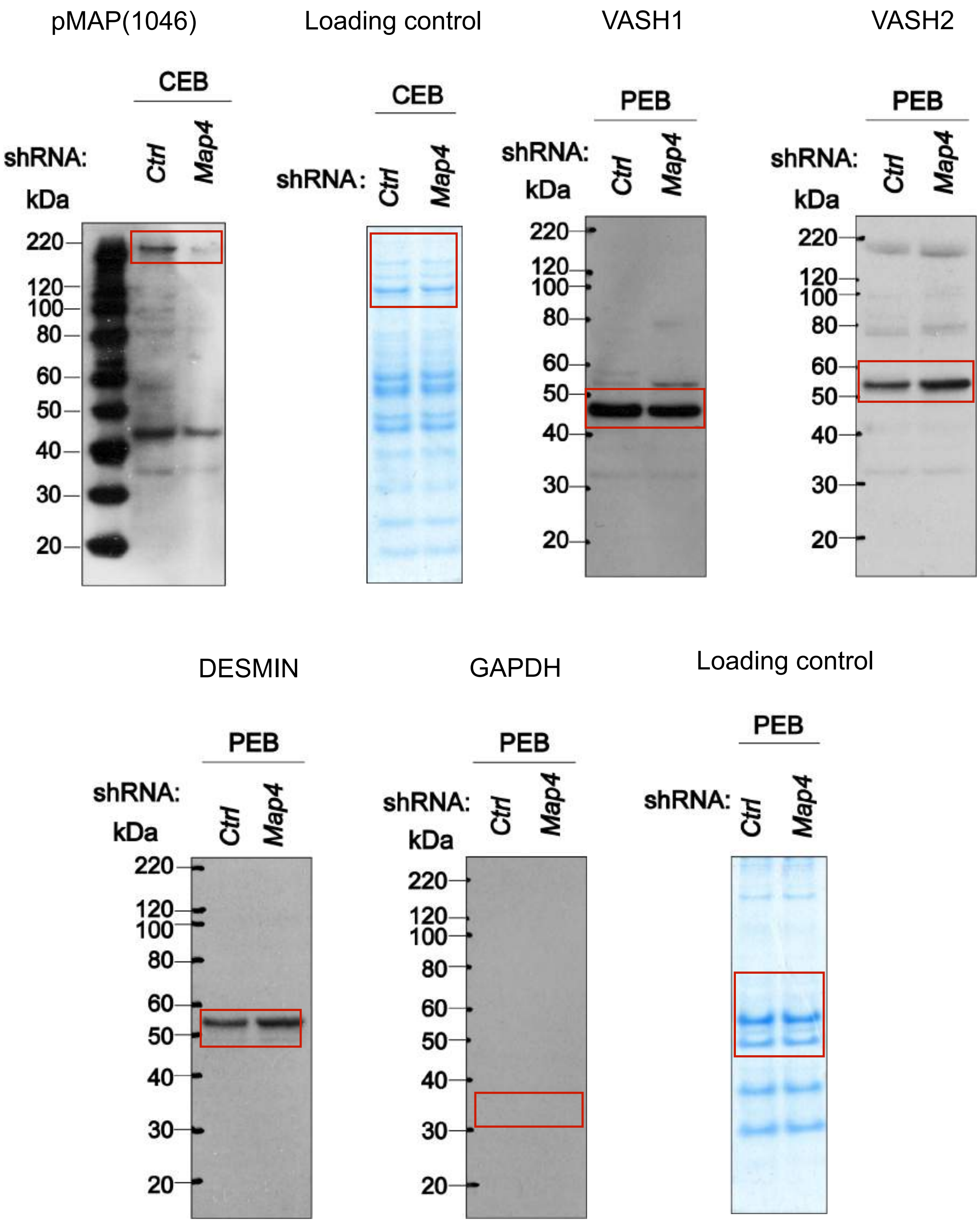

Extended Data Fig. 8a

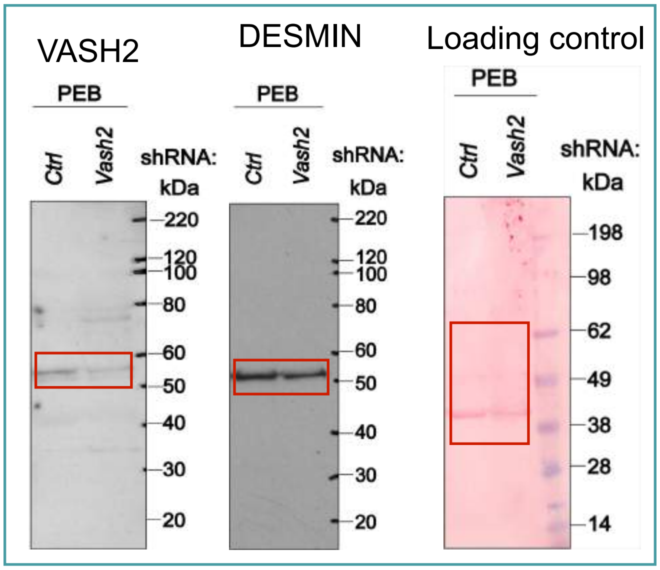

Extended Data Fig. 8b

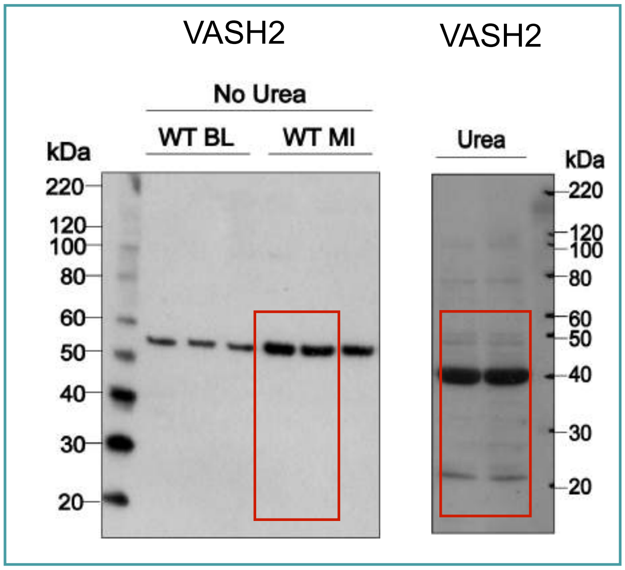

Extended Data Fig. 8e

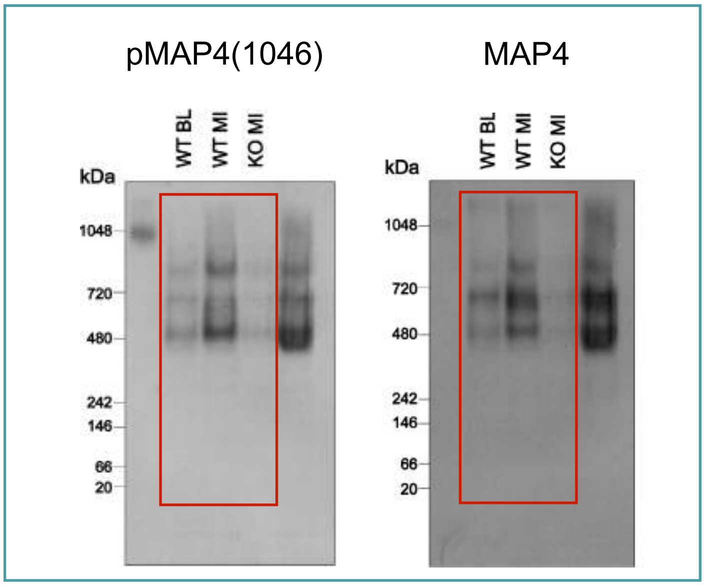

Extended Data Fig. 8f

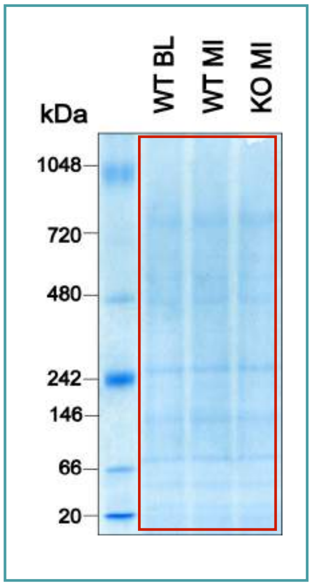

Extended Data Fig. 8g

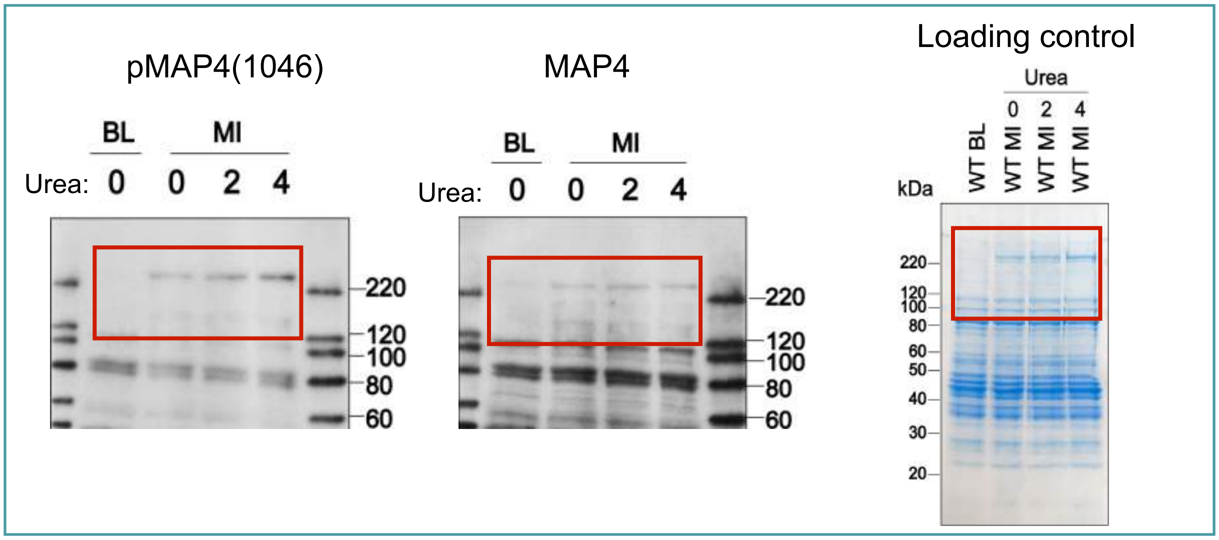

Extended Data Fig. 8h

VASH2

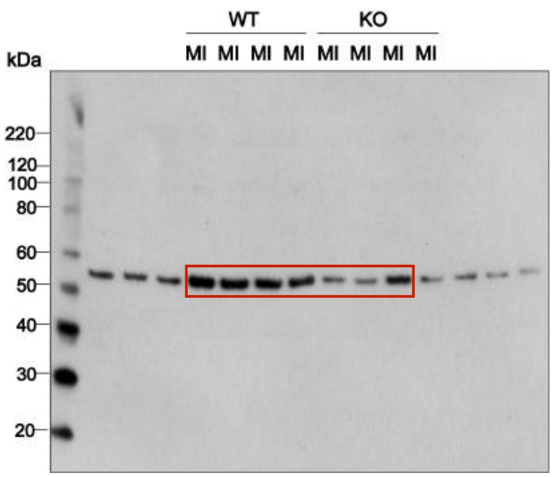

DESMIN

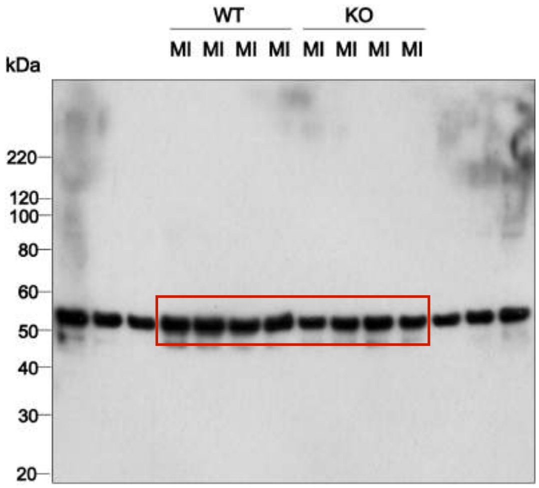

Loading control

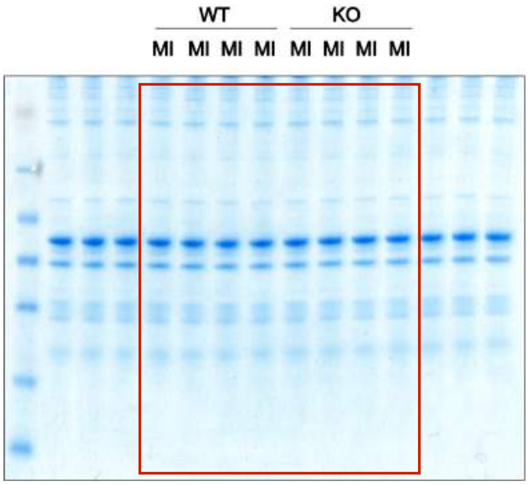

Extended Data Fig. 9a

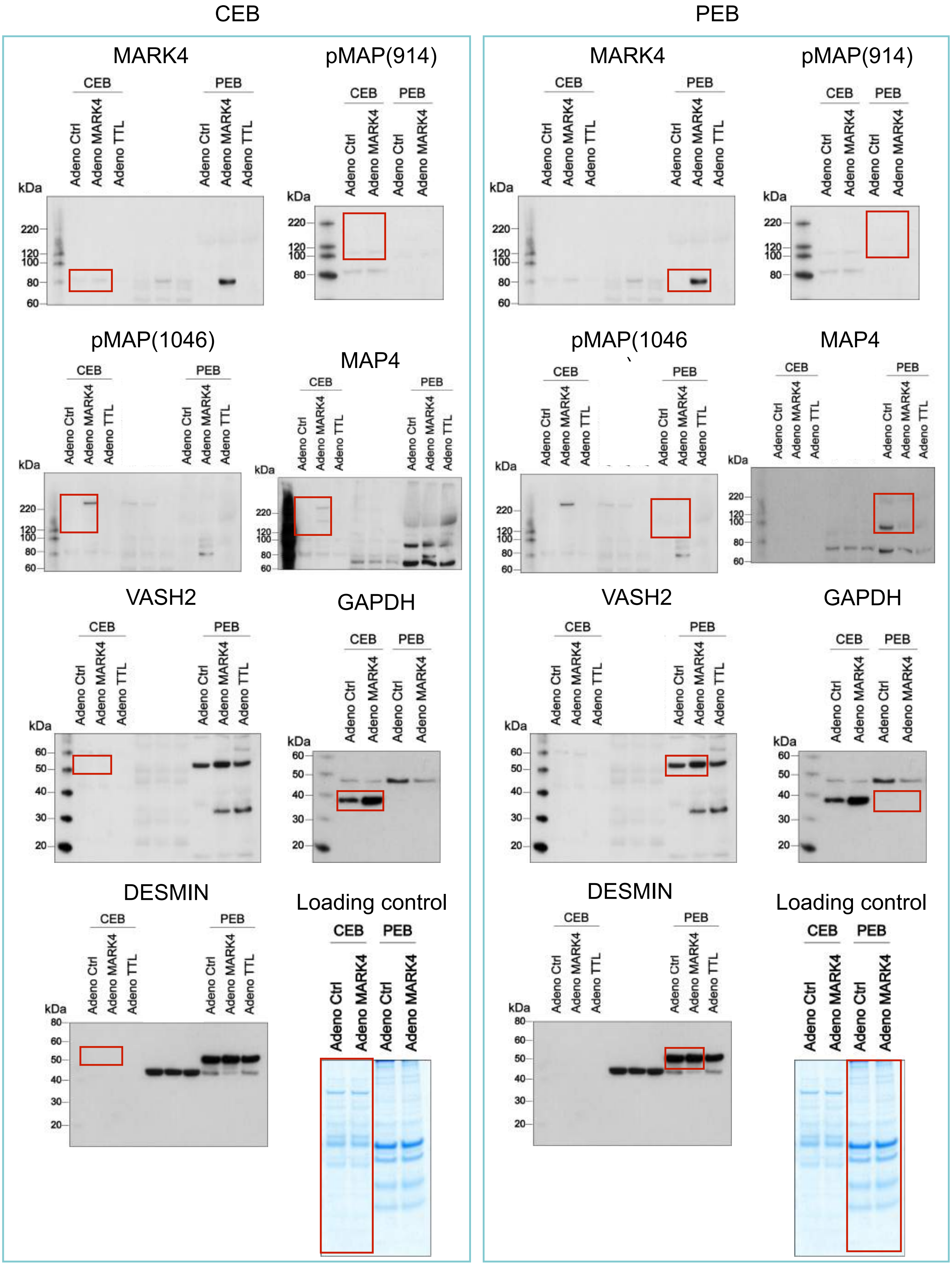

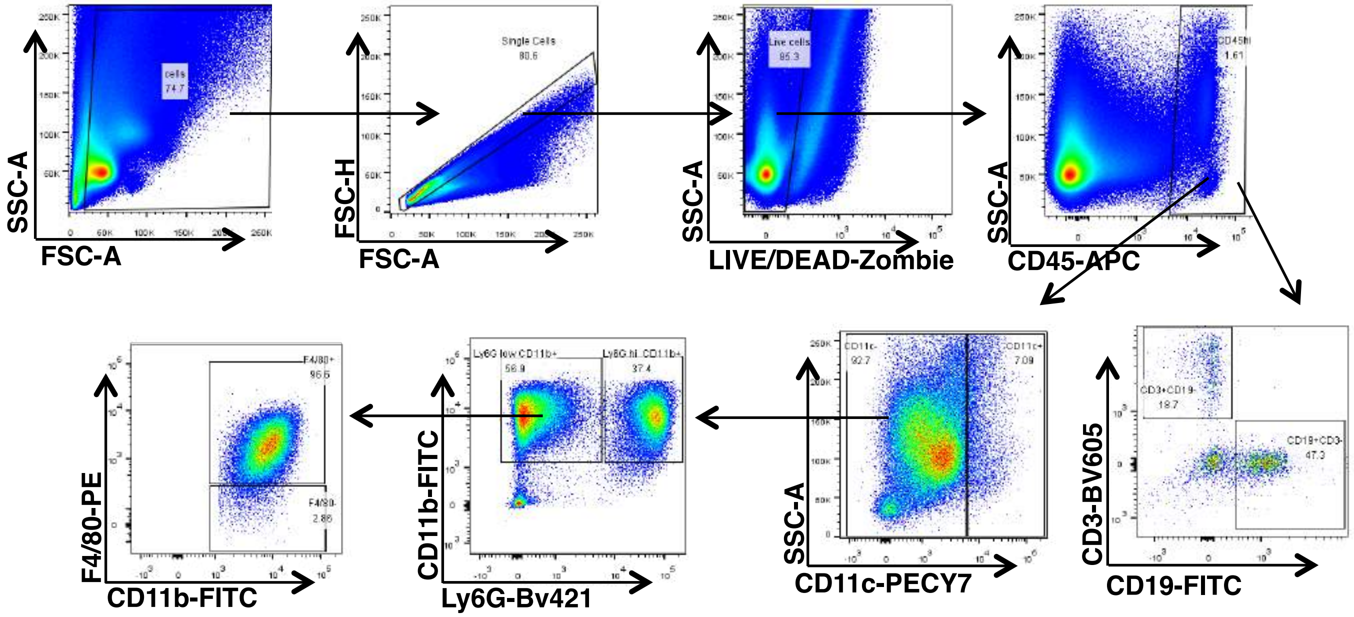

Supplementary Figure 2. Gating strategy for infiltrating immune cells.

Cardiac neutrophils were defined as $CD45^{+}CD11c^{low}CD11b^{+}Ly6G^{high}$; cardiac macrophages were defined as $CD45^{+}CD11c^{low}$ $CD11b^{+}Ly6G^{low}F4/80^{+}$; Cardiac $CD45^{+}CD11c^{high}$ include dendritic cells (DCs) and macrophages; B cells were defined as $CD45^{+}CD19^{+}CD3^{-}$ and T cells were defined as $CD45^{+}CD3^{+}CD19^{-}$.
