# Supplementary material for: MARK4 controls ischaemic heart failure through microtubule detyrosination
Source: Nature. Author manuscript; Available in PMC 2021 Dec 31. (PMC7612144; doi:10.1038/s41586-021-03573-5)
Supplement: EMS140663_Sup_info [file EMS140663-supplement-EMS140663_Sup_info.pdf]

Supplementary information guide

The supplementary information of this manuscript includes Supplementary Figures (one PDF file) and Supplementary Tables (two Excel files).

1. Supplementary Figures

Supplementary Figure 1 (page 1-15)
Immunoblots, and loading controls (Coomassie stained gels and Ponceau S stained membranes) associated with the data presented in the Figures and Extended Data Figures.

Supplementary Figure 2 (page 16)
Gating strategy for infiltrating immune cells post-myocardial infarction.

2. Supplementary Tables

Supplementary Table 1
The effect of MARK4 deficiency on cardiac parameters after myocardial infarction (MI). M-mode echocardiography was acquired at baseline, and week 1 (W1), week 2 (W2), week 4 (W4) post-myocardial infarction (MI). All statistical tests were Two-way ANOVA with Bonferroni post-hoc correction for multiple comparisons. All data are mean±s.e.m. $P>0.05$ is considered statistically non-significant.

Supplementary Table 2
The effect of MARK4 deficiency on immune cells infiltrating the hearts after myocardial infarction (MI). Immune cells were collected from hearts at day 3 post-MI or post-sham surgery. Data were analyzed using unpaired *t*-test (two-tailed), and presented as mean±s.e.m. $P>0.05$ is considered statistically non-significant.
